# Yap1 safeguards mouse embryonic stem cells from excessive apoptosis during differentiation

**DOI:** 10.7554/eLife.40167

**Published:** 2018-12-18

**Authors:** Lucy LeBlanc, Bum-Kyu Lee, Andy C Yu, Mijeong Kim, Aparna V Kambhampati, Shannon M Dupont, Davide Seruggia, Byoung U Ryu, Stuart H Orkin, Jonghwan Kim

**Affiliations:** 1 Department of Molecular BiosciencesThe University of Texas at AustinAustinUnited States; 2 Institute for Cellular and Molecular Biology, Center for Systems and Synthetic BiologyThe University of Texas at AustinAustinUnited States; 3 Division of Hematology/OncologyBoston Children’s HospitalBostonUnited States; 4 Howard Hughes Medical InstituteBostonUnited States; 5 Department of Pediatric OncologyDana-Farber Cancer Institute (DFCI)BostonUnited States; 6 Harvard Stem Cell InstituteHarvard Medical SchoolBostonUnited States; California Institute of TechnologyUnited States; California Institute of TechnologyUnited States

**Keywords:** Yap1, apoptosis, embryonic stem cells, differentiation, Mouse

## Abstract

Approximately, 30% of embryonic stem cells (ESCs) die after exiting self-renewal, but regulators of this process are not well known. Yap1 is a Hippo pathway transcriptional effector that plays numerous roles in development and cancer. However, its functions in ESC differentiation remain poorly characterized. We first reveal that ESCs lacking Yap1 experience massive cell death upon the exit from self-renewal. We subsequently show that Yap1 contextually protects differentiating, but not self-renewing, ESC from hyperactivation of the apoptotic cascade. Mechanistically, Yap1 strongly activates anti-apoptotic genes via *cis-*regulatory elements while mildly suppressing pro-apoptotic genes, which moderates the level of mitochondrial priming that occurs during differentiation. Individually modulating the expression of single apoptosis-related genes targeted by Yap1 is sufficient to augment or hinder survival during differentiation. Our demonstration of the context-dependent pro-survival functions of Yap1 during ESC differentiation contributes to our understanding of the balance between survival and death during cell fate changes.

## Introduction

Yap1 regulates genes involved in many cellular functions, including proliferation, organ size control, and tumorigenesis (Ehmer and Sage, 2016; Hansen et al., 2015; Huang et al., 2005). When Hippo signaling is active, kinases Lats1/2 phosphorylate Yap1, leading to cytoplasmic sequestration (Hao et al., 2008). When Hippo signaling is inactive, Yap1 translocates to the nucleus to co-activate or co-repress numerous target genes with interacting partner proteins such as Tead factors (Kim et al., 2015; Stein et al., 2015).

Previous research indicated that, in mouse embryonic stem cells (ESCs), nuclear translocation of Yap1 occurs shortly after withdrawal of leukemia inhibitory factor (LIF), a cytokine that maintains self-renewal, and that depletion of Yap1 inhibits differentiation, whereas overexpression (OE) of Yap1 stimulates differentiation (Chung et al., 2016). Deletion of Yap1 leads to embryonic lethality by E10.5 although the downstream mechanism remains poorly characterized (Morin-Kensicki et al., 2006). Additionally, whether Yap1 has any other roles during ESC differentiation and early development remains unclear.

Apoptosis influences numerous biological processes, including development, differentiation, and infection (Fuchs and Steller, 2011; Meier et al., 2000). A previous study has reported that withdrawal of LIF causes the death of 30% or more of ESCs (Bashamboo et al., 2006; Duval et al., 2000), and around 30% of human ESCs are also annexin V positive when they exit from self-renewal (Dravid et al., 2005). A proposed function of apoptosis during ESC differentiation is to cull cells that fail to exit self-renewal, thus promoting efficient differentiation (Wang et al., 2015). This process is not limited to ESCs, as defective cells are executed during human neural progenitor differentiation as well (Jaeger et al., 2015), and apoptosis eliminates self-reactive and non-reactive lymphocytes during T and B cell differentiation (Francelin, 2011; Nemazee, 2017; Opferman, 2008).

This process must be finely tuned to ensure efficient changes in cell identity without excessive loss of cell viability. However, mechanisms that regulate the balance between survival and death during ESC differentiation remain insufficiently characterized. Here, we find that Yap1 attenuates mitochondrial apoptosis during ESC differentiation, primarily by upregulating anti-apoptotic factors, such as Bcl-2, Bcl-xL (*Bcl2l1*), and Mcl-1, through direct transcriptional regulation. Mouse ESCs lacking Yap1 have no defect in survival in self-renewing conditions. However, just after the exit from self-renewal, we find that Yap1 knockout (KO) cells develop a high degree of mitochondrial priming that precedes elevated rates of apoptosis. OE of anti-apoptotic factors or repression of pro-apoptotic factors in Yap1 KO cells rescues this enhanced rate of cell death during differentiation. This collectively suggests that Yap1 is critical for ESC survival in a context-dependent manner, advancing our understanding of regulation of cell death during changes in cell identity.

## Results

### Genetic ablation of *Yap1* intensifies caspase-dependent cell death during ESC differentiation

**Figure 1.**
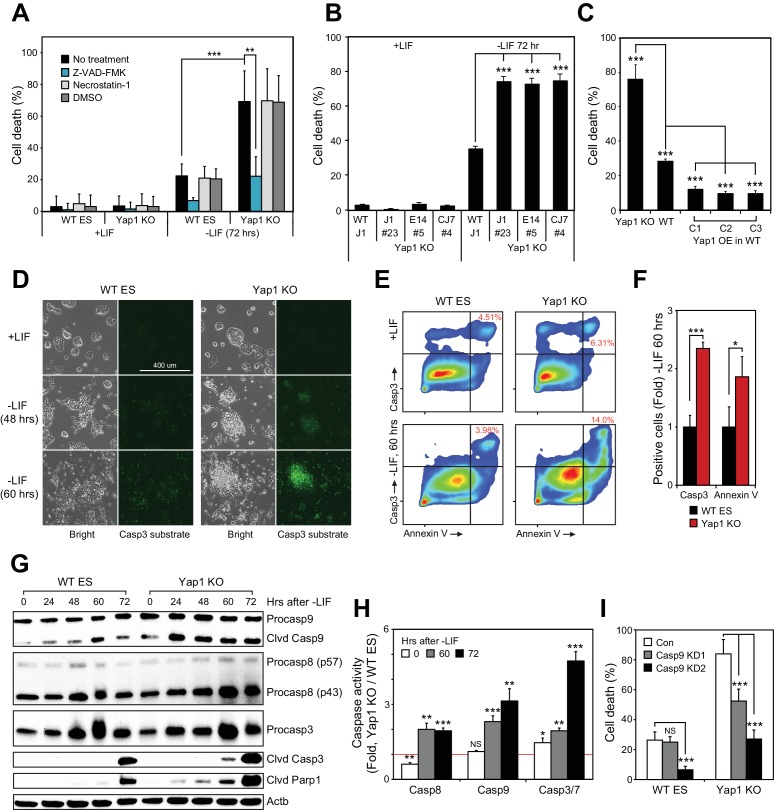
Loss of Yap1 substantially increases apoptosis during ESC differentiation. (**A**) Lactate dehydrogenase (LDH) assay of WT and Yap1 KO ESCs in ±LIF. Cells were treated with either Z-VAD-FMK (Z-VAD), necrostatin-1, DMSO, or no treatment. Values were normalized to wells that had been lysed completely. (**B**) LDH assay measuring cell death after Yap1 KO in three different ESC lines during differentiation (72 hr) or self-renewal. (**C**) LDH assay measuring cell death in Yap1 KO, WT, and three different stable FLAG-Bio (FB) Yap1 overexpression cell lines during differentiation (72 hr). (**D**) Representative brightfield and fluorescence microscopy images of WT and Yap1 KO ESCs incubated with NucView 488 Casp3 substrate at the indicated times after LIF withdrawal. (**E**) Representative flow cytometry density plots of WT and Yap1 KO ESCs detecting fluorescent signal from annexin-V (conjugated to CF594) and NucView 488 reagent during differentiation (60 hr). (**F**) Fold enrichment of annexin-V and active Casp3-positive Yap1 KO vs. WT ESCs according to flow cytometry. (**G**) Immunoblot of Casp9, Casp8, Casp3, cleaved Casp3, and cleaved Parp1 in WT and Yap1 KO cells during differentiation. β-actin was used as a loading control. (**H**) Luminescent assay of caspase activity in Yap1 KO vs. WT ESCs in ±LIF media. (**I**) LDH assay of WT and Yap1 KO cells ± KD of Casp9 during differentiation (72 hr). All data are expressed as mean ±standard deviation (n = 4 independent samples for LDH assays and n = 3 for other experiments). Two sample two-tailed t-test compared to WT or whatever is specified on the y-axis: *=0.05 > P > 0.01. **=0.01 > P > 0.001. ***=0.001 ≥ P.

To determine context-specific roles of Yap1, we attempted to differentiate J1 ESCs in which *Yap1* had been deleted via CRISPR/Cas9 in KO clones established in our previous publication (Figure 1—figure supplement 1A). While ~30% cell death was observed from wild-type (WT) cells as previously reported (Bashamboo et al., 2006), cell death was dramatically higher (up to >70%) in Yap1 KO cells 72 hr after LIF withdrawal (Figure 1A and Figure 1—figure supplement 1B). In both cases, cell death was substantially reduced after supplementation with Z-VAD-FMK (zVAD), a pan-caspase inhibitor, but not with necrostatin-1, which blocks necroptosis. Undifferentiated cells had extremely low rates of cell death regardless of genotype (Figure 1A). An additional J1 Yap1 KO clone as well as Yap1 KO clones established in the CJ7 and E14 ESC lines (Figure 1—figure supplement 1C) also experienced drastically heightened cell death during differentiation, but not self-renewal (Figure 1B). Furthermore, depletion of *Yap1* using shRNA-mediated knockdown (KD) dose-dependently increased cell death during differentiation (Figure 1—figure supplement 1D and E). Finally, measuring cell death in stable Yap1 OE cell lines (Figure 1-figure supplement F) reduced cell death to a mere ~10% during differentiation (Figure 1C). Thus, Yap1 is key for survival during ESC differentiation, and ablation of *Yap1* specifically exacerbates apoptosis.

**Figure 1—figure supplement 1.**
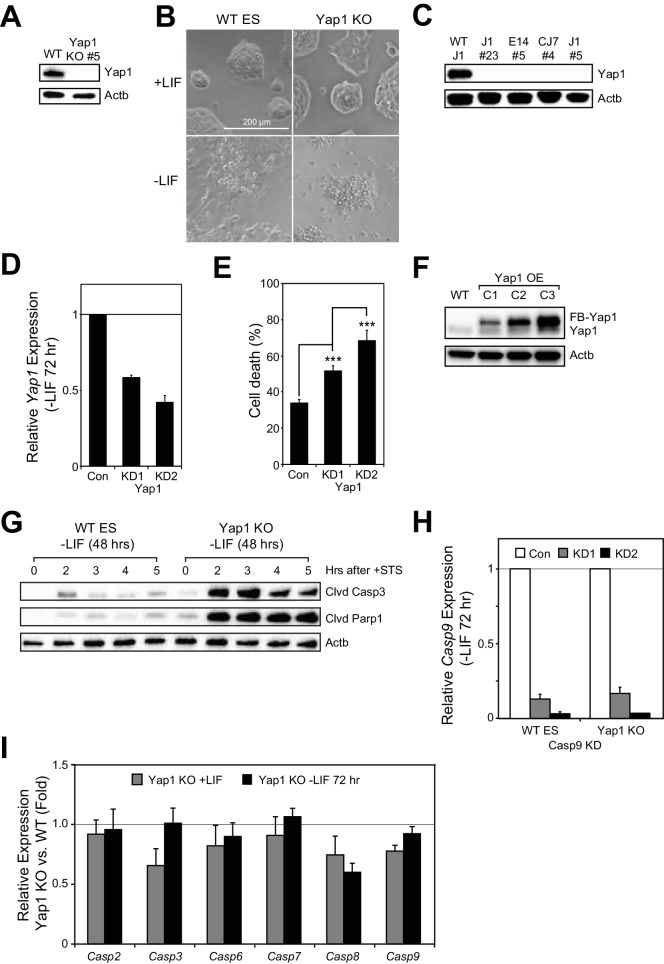
Yap1 expression in KO/KD/OE cell lines, STS sensitivity, and caspase expression during ES cell differentiation. (**A**) Immunoblot of Yap1 to verify knockout in Yap1 KO cells. β-actin was used as a loading control. (**B**) Representative brightfield microscopy images of WT and Yap1 KO ES cells in ±LIF. Scale bar, 200 μm. (**C**) Immunoblot of Yap1 to verify knockout of Yap1 in three different ESC lines (J1, E14, and CJ7). β-actin was used as a loading control. J1 clone #5 was used as a positive control for knockout. (**D**) RT-qPCR measuring the expression of Yap1 after lentiviral shRNA-mediated Yap1 KD in differentiating WT ESCs (-LIF 72 hr). (**E**) LDH assay measuring cell death of Yap1 KD vs. control KD cells during differentiation (-LIF 72 hr). (**F**) Immunoblot of Yap1 to verify stable overexpression (OE) of FLAG-Bio-Yap1 in three different clones compared to WT ESCs. β-actin was used as a loading control. (**G**) Immunoblot of cleaved Casp3 and cleaved Parp1 in WT and Yap1 KO cells that had been treated with 1 μM STS for the indicated number of hours during differentiation (treatment started 43–48 hr after withdrawal of LIF depending on the length of STS treatment). (**H**) RT-qPCR measuring the expression of Casp9 upon shRNA-mediated lentiviral KD in WT and Yap1 KO cells during differentiation (72 hr) relative to empty vector KD. (**I**) RT-qPCR measuring the expression of Casp2, Casp3, Casp6, Casp7, Casp8, and Casp9 in Yap1 KO cells compared to WT cells in ±LIF. All data are expressed as mean ±standard deviation (n = 3 independent samples). Two sample two-tailed t-test compared to WT or whatever is specified on the y-axis: *=0.05 > P > 0.01. **=0.01 > P > 0.001. ***=0.001 ≥ P.

### Loss of Yap1 leads to caspase hyperactivation during differentiation

During apoptosis, initiator caspases 8 (Casp8) and 9 (Casp9) are activated first, either by death receptors or mitochondrial outer membrane permeabilization, respectively (Bao and Shi, 2007). They then cleave executioner caspases such as caspase-3 (Casp3), which then cleave hundreds of downstream targets in the cell that result in its death, including Parp1 (Fischer et al., 2003). Treatment of ESCs with NucView 488 enabled live visualization of active Casp3. In undifferentiated ESCs, Casp3 activation was rare in both WT and KO cells, but the proportion of cells with active Casp3 increased visibly after LIF withdrawal as a function of time (Figure 1D). Notably, a far greater proportion of Yap1 KO cells than WT cells possessed active Casp3 by 60 hr. Then, we performed flow cytometry to quantify active Casp3 as well as externalized phosphatidylserine. Both the relative proportion of Casp3 positive cells and the fluorescent intensity of the Casp3 substrate fluorescent probe were higher in Yap1 KO differentiating ESCs (dESCs), and this was correlated with an increased proportion of annexin V positive cells (Figure 1E and F). Immunoblot analysis confirmed faster and more intense cleavage of Casp9, Casp3, and Parp1 in Yap1 KO cells during differentiation (Figure 1G). To determine whether Yap1 KO dESCs were more sensitive to exogenous apoptosis-inducing stimuli, we treated dESCs with staurosporine (STS), a high-affinity, non-specific kinase inhibitor that has long been used to dissect the induction of intrinsic apoptosis in a myriad of cellular contexts (Belmokhtar et al., 2001; Preta and Fadeel, 2012; Xu et al., 2015). This treatment induced faster and more drastic activation of Casp3 and Parp1 in Yap1 KO than in WT dESCs as quickly as two hours after addition, reflecting a vastly heightened sensitivity to apoptosis-inducing stress (Figure 1—figure supplement 1G).

Next, we quantified caspase activity using a luminogenic substrate. By 60 hr after LIF removal, all caspases tested were approximately two-fold more active in Yap1 KO cells than in WT (Figure 1H). These observations demonstrate that lack of Yap1 accelerates and intensifies caspase activation during differentiation. We decided to dissect which part of the apoptotic pathway is affected first by loss of Yap1. Though Casp8 activity is elevated in Yap1 KO cells, we did not detect substantial differences in cell death after Casp8 KD (data not shown), so we decided to target Casp9 with two different shRNAs (Figure 1—figure supplement 1H). As expected, KD of Casp9 reduced cell death during differentiation, and this was particularly stark for Yap1 KO cells, where cell death was reduced to WT levels without Casp9 KD (Figure 1I). This implied that the abnormally high rates of apoptosis in Yap1 KO cells are sustained by heightened Casp9 activation. We observed that mRNA expression of caspases was relatively equal between Yap1 KO cells and WT cells during differentiation (Figure 1—figure supplement 1I). Additionally, protein levels of Casp3 showed similar fluctuations in dESCs for both WT and KO cells; although Casp8 and Casp9 were elevated in Yap1 KO cells (Figure 1G). However, since caspase activity is strongly activated by cleavage (Hu et al., 2013), we speculated that Yap1 may regulate other factors that indirectly affect the rate of caspase cleavage.

### Yap1 protects against apoptosis regardless of differentiation method and acts directly after the exit from self-renewal

**Figure 2.**
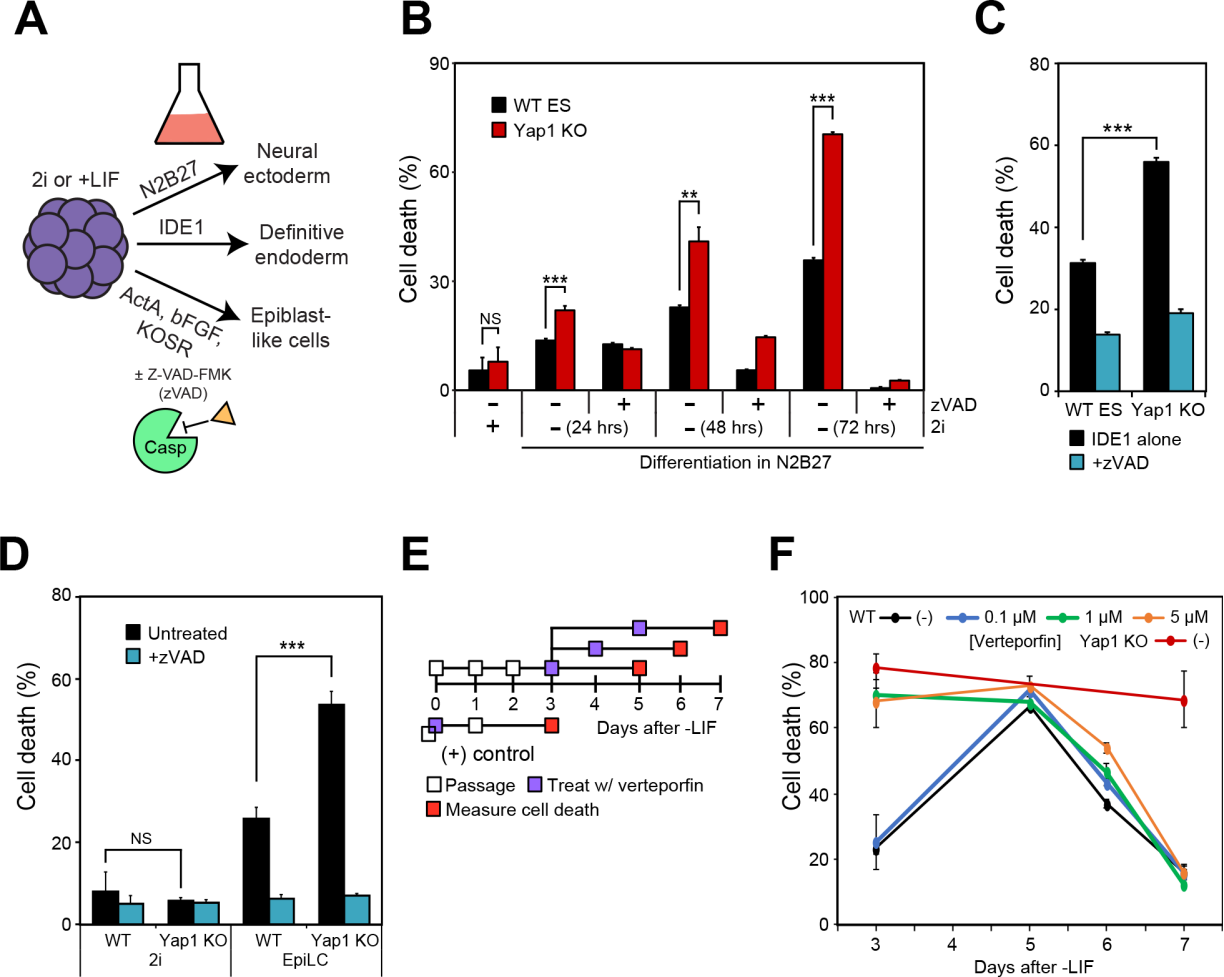
Loss of Yap1 augments apoptosis in several differentiation conditions, but its role is largely restricted to the exit from self-renewal. (**A**) Schematic of 3 differentiation protocols (ectoderm, endoderm, and epiblast) used in Figures 2, 3 and 5. (**B**) LDH assay of WT and Yap1 KO ESCs in N2B27 with or without 2i and Z-VAD. (**C**) LDH assay of WT and Yap1 KO ESCs in low serum DMEM supplemented with IDE1 ±Z VAD (48 hr). (**D**) LDH assay of ESC towards EpiLC conversion in WT and Yap1 KO ESCs (72 hr). (**E**) Schematic of verteporfin (vert) treatment timings during late and early differentiation in WT ESCs in -LIF. (**F**) Timecourse LDH assay of verteporfin-treated dESCs at the indicated timepoints along with positive controls (treatment with verteporfin just after -LIF as well as untreated Yap1 KO ESCs, the latter of which are n = 8). All data are expressed as mean ±standard deviation (n = 4 independent samples unless otherwise stated). Two sample two-tailed t-test compared to WT or whatever is specified on the y-axis: *=0.05 > P > 0.01. **=0.01 > P > 0.001. ***=0.001 ≥ P.

To determine whether the roles of Yap1 are either specific to LIF withdrawal or broadly applicable to the exit from self-renewal in different conditions, we utilized alternate differentiation methods (Figure 2A). Utilizing N2B27 medium (neural ectoderm fate) or low serum DMEM supplemented with IDE1 (definitive endoderm fate) (Borowiak et al., 2009), we again observed that dESCs without Yap1 experienced much higher rates of cell death compared to WT cells, which could be rescued by zVAD (Figure 2B and C). We verified by RT-qPCR that N2B27 medium indeed induced neural ectoderm marker expression (Figure 2—figure supplement 1A) whereas IDE1 treatment induced endoderm marker expression (Figure 2—figure supplement 1B), as well as repression of *Nanog*, an ESC self-renewal marker.

**Figure 2—figure supplement 1.**
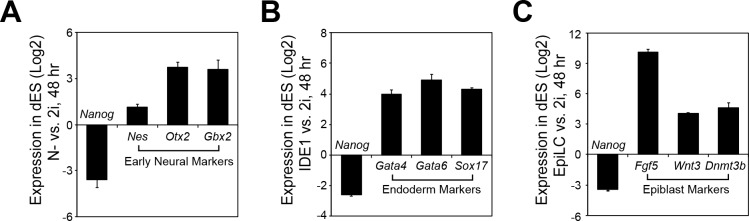
Lineage marker expression during various differentiation methods. (**A**) RT-qPCR measuring the expression of neural ectoderm markers *Nes*, *Otx2*, and *Gbx2*, as well as pluripotency marker *Nanog*, in cells that had been differentiating in N2B27 neural differentiation media for 48 hr. Expression was normalized to ESCs grown in 2i. (**B**) RT-qPCR measuring the expression of endoderm markers *Gata4*, *Gata6*, and *Sox17*, as well as pluripotency marker *Nanog*, in cells that had been differentiating in low serum DMEM supplemented with 5 μM IDE1 for 48 hr. Expression was normalized to ESCs grown in 2i. (**C**) RT-qPCR measuring the expression of epiblast markers *Fgf5*, *Wnt3*, and *Dnmt3b*, as well as pluripotency marker *Nanog*, in cells that had been differentiating in EpiLC conditions for 48 hr. Expression was normalized to ESCs grown in 2i. All data are expressed as mean ±standard deviation (n = 3 independent samples).

We also induced differentiation towards epiblast-like cells (EpiLCs) to mimic early embryo development in vitro; whereas mESCs are equivalent to the inner cell mass of the blastocyst at E3.5–4.5, EpiLCs represent the next developmental stage, the E5.5–6.0 epiblast (Hayashi et al., 2011). We confirmed repression of *Nanog* and upregulation of EpiLC-specific markers (Figure 2—figure supplement 1C). As expected, EpiLCs lacking Yap1 underwent substantially higher cell death than WT by d3, and zVAD reduced cell death in both genotypes to the low, basal rates experienced in 2i media (Figure 2D). Finally, we used a well-characterized inhibitor of Yap1, verteporfin (Brodowska et al., 2014), to investigate Yap1’s role during late -LIF differentiation (Figure 2E). While treatment with as low as 1 μM verteporfin before the exit from self-renewal phenocopied Yap1 KO, treatment during late differentiation had more modest effects on cell death, and treated cells had death rates nearly identical to untreated by d7 (Figure 2F). Together, these data suggest that loss of *Yap1* increases rates of apoptosis in ESCs directly after the exit from self-renewal, regardless of the ultimate lineage those ESCs are destined for.

### Yap1 modulates the expression of apoptosis-related genes during differentiation

**Figure 3.**
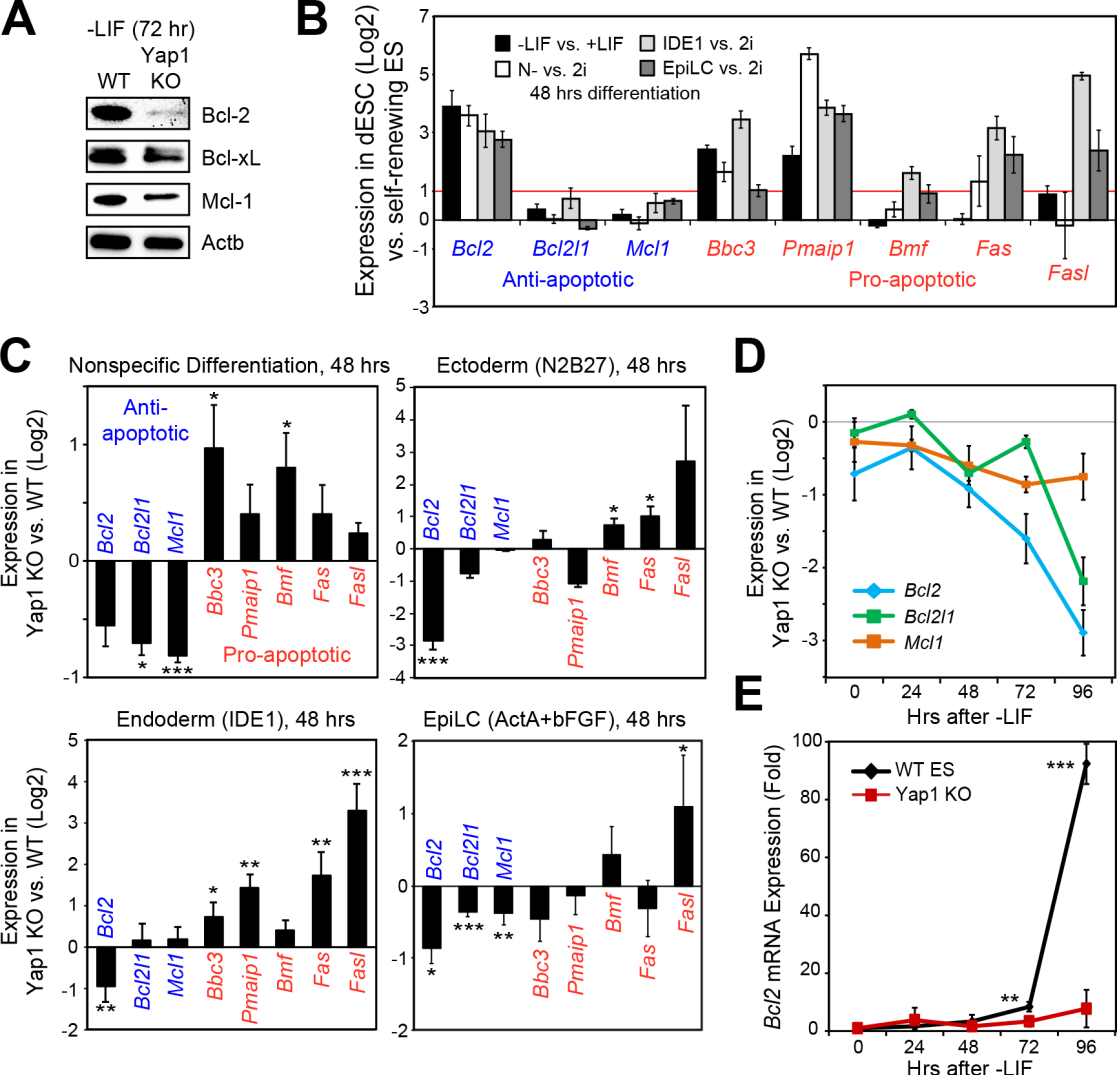
Loss of Yap1 leads to abnormal expression of apoptosis-related genes. (**A**) Immunoblot of Bcl-2, Bcl-xL, and Mcl-1 in WT and Yap1 KO cells in -LIF after 72 hr of differentiation. (**B**) RT-qPCR measuring the expression of anti-apoptotic (blue) and pro-apoptotic (red) genes in WT ESCs cultured in the indicated differentiation conditions (all at 48 hr) normalized to their respective self-renewal conditions. (**C**) RT-qPCR measuring the expression of anti- and pro-apoptotic genes in Yap1 KO vs. WT cells (log_2_) in various differentiation conditions (all at 48 hr). (**D**) RT-qPCR measuring the expression of *Bcl2*, *Bcl2l1*, and *Mcl1* in Yap1 KO cells vs. WT cells during differentiation (timecourse). (**E**) RT-qPCR measuring the expression of *Bcl2* in WT and Yap1 KO cells during differentiation (timecourse) relative to +LIF. All data are expressed as mean ±standard deviation (n = 3 independent samples unless otherwise stated). Two sample two-tailed t-test compared to WT or whatever is specified on the y-axis: *=0.05 > P > 0.01. **=0.01 > P > 0.001. ***=0.001 ≥ P. 10.7554/eLife.40167.008Figure 3—source data 1.Data used in Figure 3—figure supplement 1D and E.

Following our deduction that Casp9 hyperactivation distinguishes Yap1 KO dESCs from WT dESCs and that lack of *Yap1* enhances apoptosis in several differentiation conditions, we examined the expression of anti- and pro-apoptotic genes that affect Casp9 activation. After 72 hr of LIF withdrawal, we detected a deficiency in three key anti-apoptotic proteins (Bcl-2, Bcl-xL, and Mcl-1) in Yap1 KO cells by immunoblot (Figure 3A). Immunocytochemistry confirmed reduced expression of Bcl-2 and Mcl-1 in Yap1 KO dESCs compared to WT, as well as lower mitochondrial content as measured by MitoTracker dye (Figure 3—figure supplement 1A and B); as expected, Bcl-2 and Mcl-1 strongly colocalized with the mitochondria (weighted colocalization coefficient for all samples ~ 0.7–0.9). We then investigated the significance of this expression defect in the context of what normally happens during differentiation. In WT ESCs, we found that *Bcl2* was strongly upregulated in all differentiation conditions tested; pro-apoptotic genes such as Puma (*Bbc3*) and Noxa (*Pmaip1*) were also activated, whereas *Bcl2l1* and *Mcl1* either stayed constant or were weakly upregulated (Figure 3B). Comparing Yap1 KO cells to WT ESCs, by d2, we found a general trend for decreased anti-apoptotic gene expression (most consistently *Bcl2*) and increased pro-apoptotic gene expression (Figure 3C). This defect worsened over time in -LIF (Figure 3D) and was particularly stark for *Bcl2*, which was upregulated as much as 80 to 100-fold by 96 hr in WT ESCs upon differentiation (Figure 3E).

**Figure 3—figure supplement 1.**
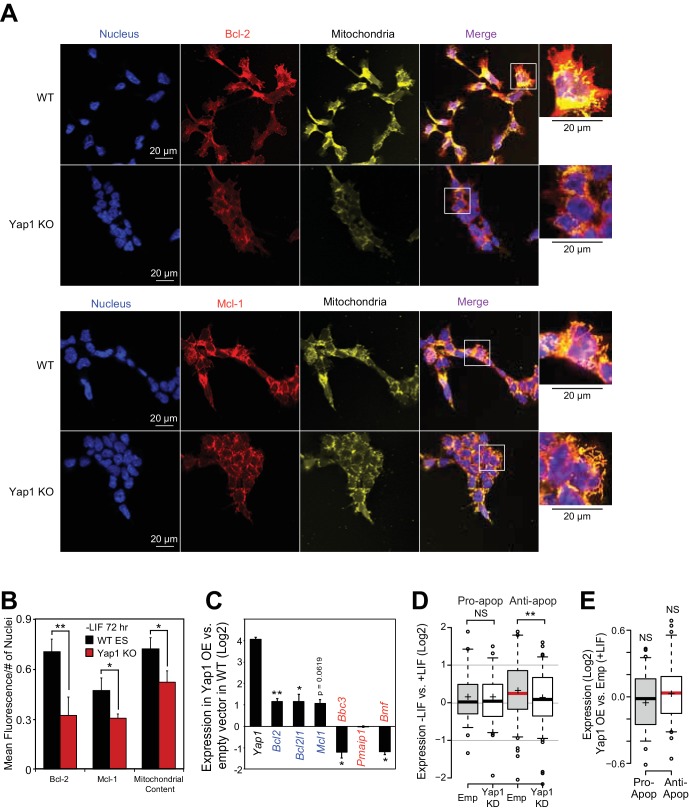
Depletion or loss of Yap1 leads to dysregulation of apoptosis-related genes. (**A**) Representative confocal images (63X oil objective) of immunocytochemistry of WT and Yap1 KO ESCs in -LIF (72 hr). Blue represents the nucleus, red represents Bcl-2 (top 6) or Mcl-1 (bottom 6), and yellow represents mitochondria. White squares indicate location of zoom images. Scale bar, 20 μm. (**B**) Quantification of fluorescence corresponding to Bcl-2, Mcl-1, and mitochondria using ImageJ, normalized to the number of nuclei in each view as stained by NucBlue. (**C**) RT-qPCR of WT ESCs with transient OE of Yap1 (48 hr) in -LIF (72 hr) normalized to empty vector. Blue indicates anti-apoptotic genes, red indicates pro-apoptotic genes. (**D**) Boxplots of expression of pro-apoptotic genes and anti-apoptotic genes in Yap1 OE cells versus BirA cells (log_2_) in +LIF. Plus symbols represent the average and middle red bands represent the median. Outliers are represented by hollow circles. Significance stars indicate p-values from paired t-test. (**E**) Boxplots of expression of pro- and anti-apoptotic genes in Yap1 KD and empty KD in -LIF relative to +LIF (log2). Plus symbols represent the average and middle red bands represent the median. Outliers are represented by hollow circles. Significance stars indicate p-values from paired t-test. All data are expressed as mean ±standard deviation (n = 3 independent samples unless otherwise stated). Two sample two-tailed t-test (unless otherwise specified) compared to WT or whatever is specified on the y-axis: *=0.05 > P > 0.01. **=0.01 > P > 0.001. ***=0.001 ≥ P.

To reinforce this observation, we examined the expression of apoptosis-related genes after 2d of transient OE of Yap1 after 3d of differentiation total, and found a modest induction in *Bcl2*, *Bcl2l1*, and *Mcl1*, as well as a modest repression of *Bbc3* and *Bmf* (Figure 3—figure supplement 1C). Using RNA-seq data from a previous study (Chung et al., 2016), we found that differentiation induces the expression of a group of anti-apoptotic genes in WT cells, but this induction is debilitated after Yap1 KD (Figure 3—figure supplement 1D). Meanwhile, constitutive Yap1 OE during +LIF conditions appeared to slightly induce anti-apoptosis genes on average, though not significantly (Figure 3—figure supplement 1E). Collectively, these data show that Yap1 may function as a master regulator in proper maintenance or induction of anti-apoptotic genes (particularly *Bcl2*) during differentiation, and it may also dampen the upregulation of pro-apoptotic genes.

### Yap1 directly regulates apoptosis-related genes via transcription

**Figure 4.**
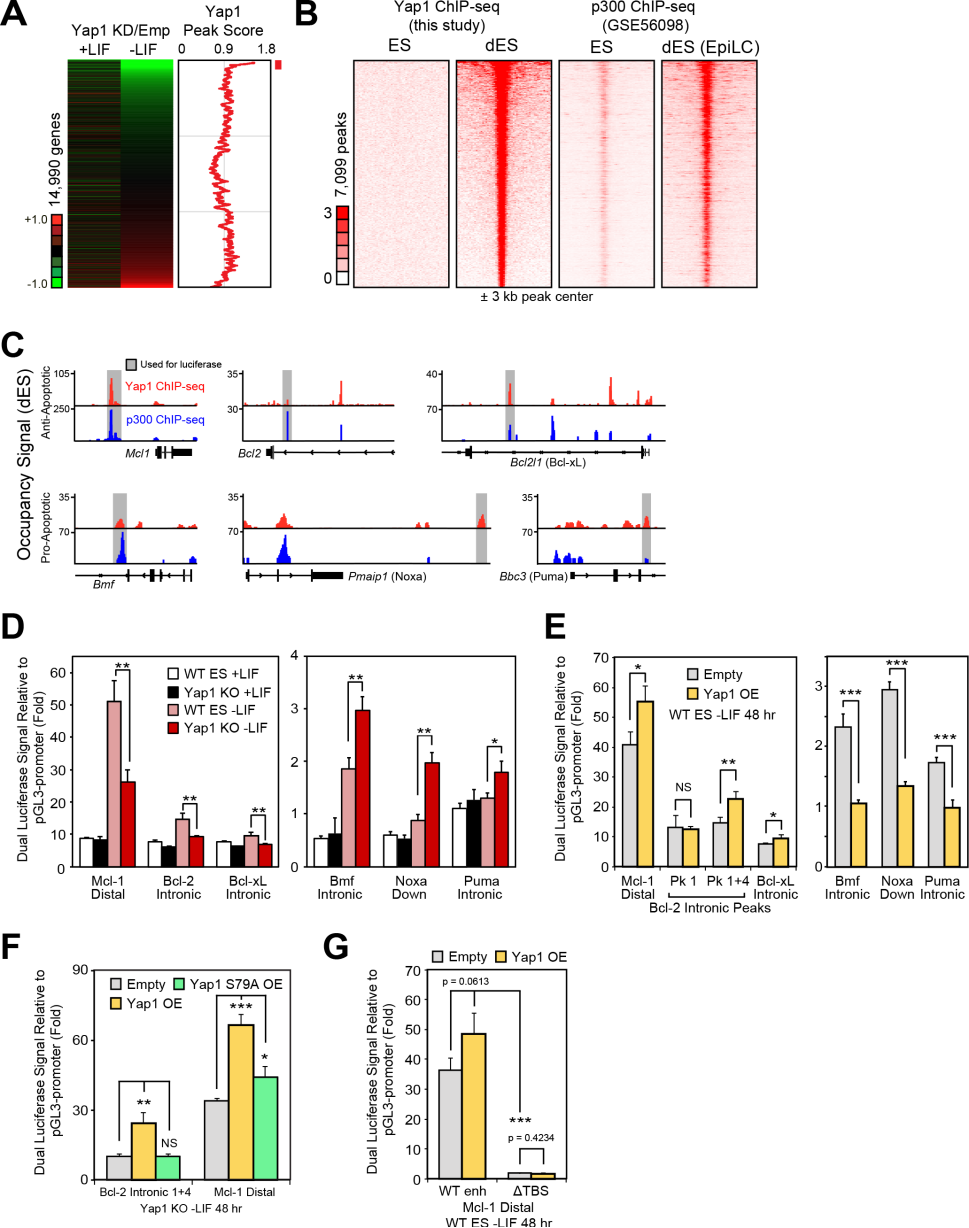
Yap1 directly regulates target apoptotic genes during differentiation. (**A**) RNA-seq heatmap (Yap1 KD/empty vector KD, in both undifferentiated and differentiating ESCs) and line graph depicting Yap1 peak score, normalized to BirA, calculated using a moving window average (window = 150). Color bar indicates extent of upregulation (red) or downregulation (green) upon Yap1 KD. (**B**) ChIP-seq peak heatmaps using coordinates centered on the top Yap1 peaks (p-value cutoff, 1e-5) in dESCs (-LIF), which are shown in the second heatmap from the left. The other heatmaps represent occupancy of Yap1 in ESCs (first) or p300 in ESCs (third) or dESCs (fourth) corresponding to Yap1 dESC peak centers ± 3 kb (bin size = 100). (**C**) Signal tracks of Yap1 (red) and p300 (blue) occupancy on apoptosis-related genes in dESCs and EpiLCs, respectively. (**E and F**) Dual luciferase assay of Yap1-occupied cis-regulatory elements from anti- and pro-apoptotic genes in (**E**) Yap1 KO and WT cells ± LIF (48 hr) or (**F**) WT cells with Yap1 or empty OE (in -LIF, 48 hr), relative to pGL3-promoter, 24 hr after transfection. (**G**) Dual luciferase assay of Bcl-2 and Mcl-1 regulatory elements in Yap1 KO cells after transfection of empty vector or vectors containing FLAG-Bio Yap1 with or without a Ser79Ala mutation. (**H**) Dual luciferase assay of Mcl-1 with a deletion of its Tead binding motif (GGAAT on the reverse strand) in WT ESCs ± Yap1 OE. All data are expressed as mean ±standard deviation (n = 3 independent samples unless otherwise stated). Two sample two-tailed t-test compared to WT or whatever is specified on the y-axis: *=0.05 > P > 0.01. **=0.01 > P > 0.001. ***=0.001 ≥ P. 10.7554/eLife.40167.011Figure 4—source data 1.Data used in Figure 4A, Figure 4—figure supplement 1A,C,D,E,I and K.

We performed ChIP-seq of Yap1 using ESCs overexpressing FLAG-Bio-Yap1 (FB-Yap1) under differentiation (-LIF, 72 hr) and self-renewal (+LIF) conditions, detecting 8453 peaks significantly enriched over the BirA control above threshold between duplicates during differentiation and only 699 peaks in +LIF, reflecting its known cytoplasmic localization during self-renewal. Many of the differentiation-related peaks were intergenic as well as in promoters (Figure 4—figure supplement 1A). Yap1 occupancy was positively correlated with degree of gene downregulation upon Yap1 KD, although some upregulated genes upon KD were associated with unusually low Yap1 occupancy (Figure 4A). By integrating data from a previous study investigating enhancer patterns at different stages of pluripotency (Buecker et al., 2014), we found that Yap1 peaks were strongly correlated with increased Ep300 (p300) occupancy during differentiation (Figure 4B). Although EpiLC differentiation induces a different cell fate than -LIF due to supplementation with activin A and bFGF, we reasoned that apoptosis-related regulation would be shared between the two conditions. We confirmed a physical interaction between Yap1 and p300 as well as one of its known cofactors, Tead4 (Chen et al., 2010), during ESC differentiation using co-immunoprecipitation (Figure 4—figure supplement 1B), consistent with known Yap1 nuclear localization in dESCs (Chung et al., 2016). Indeed, motif analysis revealed a significant enrichment of the Tead factor motif in addition to Zic3 and AP-1 complex (JunB and Fra1 (Fosl1)) motifs in the center of Yap1 peaks, whereas Esrrb (a negative control) was not found (Figure 4—figure supplement 1C and D). Finally, since p300 possesses histone acetyltransferase activity, we confirmed an increase in H3K27ac, an activating histone mark, in Yap1 peaks during differentiation (Figure 4—figure supplement 1E). Gene ontology (GO) analysis of genes bound by Yap1 and downregulated by Yap1 KD mainly yielded terms related to cell migration and motility, and regulation of cell death was also statistically significant (Figure 4—figure supplement 1F).

**Figure 4—figure supplement 1.**
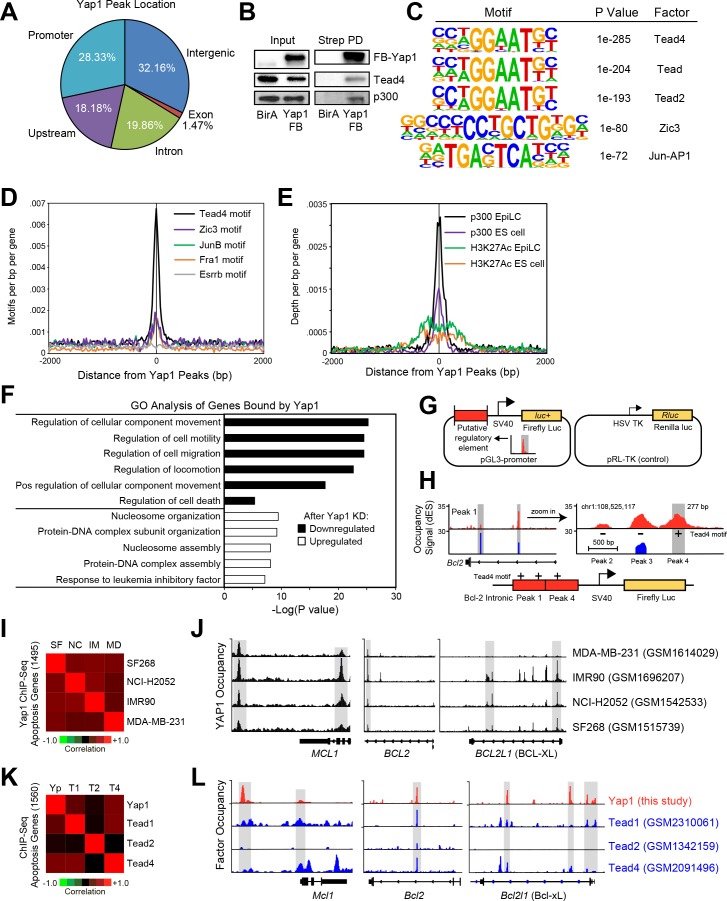
Yap1 binds to distal regulatory elements, primarily through Tead factors. (**A**) Gene feature analysis of Yap1 ChIP-seq quantifying the proportion of Yap1 peaks in promoter, intergenic, upstream, intron, or exon regions. (**B**) Co-IP followed by immunoblot of Tead4 and p300 after pull-down using magnetic streptavidin beads in Yap1 FB cells during differentiation (72 hr). (**C**) Known motif analysis of Yap1 ChIP-seq peaks using BirA ES cells as a background control. Top five motifs with the lowest p-values corresponding to known factors are presented. (**D**) Peak-centered histogram of Yap1 ChIP-seq peaks indicating the presence of the motifs of Tead4, Zic3, and AP-1 complex members JunB and Fra1 (Fosl1). Esrrb is presented as a negative control, as known motif analysis in (**C**) showed no significant enrichment of the Esrrb motif. Input represents 0.03% of total protein lysate. (**E**) Peak-centered histogram of Yap1 ChIP-seq peaks indicating p300 occupancy and H3K27ac presence in ESCs maintained in 2i and EpiLCs, which represent dESCs. (**F**) Gene ontology (GO) analysis of genes bound by Yap1 that are also upregulated (white) or downregulated (black) after Yap1 KD in -LIF (96 hr). (**G**) Schematic of dual luciferase essay using putative Yap1-responsive *cis-*regulatory elements. (**H**) Schematic of tandem Bcl-2 enhancer creation showing the location of both Yap1-occupied elements that were combined into the same construct. (**I**) Correlation heatmap of YAP1 occupancy on apoptosis-related genes in SF268 glioblastoma cells, NCI-H2052 lung mesothelioma cells, IMR90 lung fibroblasts, and MDA-MB-231 triple negative breast cancer cells. Genes that were not occupied by any factor were removed from the analysis to reduce noise. (**J**) Signal tracks of YAP1 occupancy on *MCL1*, *BCL2*, and *BCL2L1* (BCL-XL) in the cell types described in (**I**). (**K**) Correlation heatmap of occupancy of Yap1 in mouse dES cells (from this study), Tead1 in pre-B progenitor cells, Tead2 in Py2T breast cancer cells, and Tead4 in hemogenic epithelium, all on apoptosis-related genes. Genes that were not occupied by any factor were removed from the analysis to reduce noise. (**L**) Signal tracks of Yap1 (red), Tead1, Tead2, and Tead4 (all in blue) on *Mcl1*, *Bcl2*, and *Bcl2l1* (Bcl-xL) in the cell types described in (**I**).

In addition to its co-activating properties, Yap1 also acts as a co-repressor in other contexts (Kim et al., 2015), and we observed Yap1 occupancy on both anti-apoptotic and pro-apoptotic genes (Figure 4C). To characterize Yap1 target putative *cis*-regulatory elements, we performed the dual luciferase assay in Yap1 KO cells, WT cells, and cells transfected with a Yap1 OE vector using the pGL3 promoter vector (Figure 4—figure supplement 1G). In Yap1 KO cells, luciferase constructs with regulatory elements associated with *Mcl1*, *Bcl2*, or *Bcl2l1* have lower luciferase activity relative to WT cells, while regulatory elements associated with *Bmf*, *Pmaip1*, and *Bbc3* led to higher luciferase activity (Figure 4D). Meanwhile, transient OE of Yap1 led to higher luciferase activity with anti-apoptotic gene regulatory elements and lower luciferase activity with pro-apoptotic gene regulatory elements (Figure 4E). Though the initial *Bcl2* intronic regulatory element was unresponsive to Yap1 OE, combining it with another element in the same intron (Figure 4—figure supplement 1H) caused its activity to increase 2x during OE (Figure 4E).

To determine the importance of the known Yap1-Tead interaction for the function of these regulatory elements, we chose the strongest enhancers (*Mcl1* distal and *Bcl2* intronic tandem) for further testing. Transient OE of Yap1 in Yap1 KO cells rescued enhancer function to levels comparable to Yap1 OE in WT cells, whereas OE of Yap1 S79A, a mutant less capable of binding to Tead factors (Schlegelmilch et al., 2011), only mildly rescued *Mcl1*’s enhancer’s activity and failed to rescue *Bcl2*’s enhancer’s activity at all (Figure 4F). Furthermore, ablation of the Tead binding sequence (ΔTBS) from the *Mcl1* enhancer not only eliminated its Yap1 responsiveness, but also nearly abolished its enhancer activity (Figure 4G). Intriguingly, Yap1 occupancy on apoptosis-related genes seems to be relatively conserved (r =~0.5) among different human cancer cell types (Figure 4—figure supplement 1I and J). Thus, Yap1 may regulate apoptosis-related genes through conserved binding locations in both the human and mouse genome. Additionally, Yap1 peaks in mouse dESCs also correlated with Tead1 and Tead4 peaks in other mouse cell types, and signal tracks show similar occupancy patterns particularly for *Bcl2* (Figure 4—figure supplement 1K and L). Taken together, our data as well as data reanalyzed from other labs suggest that Yap1 directly regulates apoptosis-related genes.

### Loss of Yap1 contributes to heightened mitochondrial priming and dependence on anti-apoptotic proteins

Mitochondrial priming describes how close a cell is to the threshold of apoptosis and is a function of the balance between anti-apoptotic and pro-apoptotic proteins (Czabotar et al., 2013; Deng, 2017; Sarosiek et al., 2013). Since Yap1 KO cells already show higher expression of pro-apoptotic genes and lower expression of anti-apoptotic genes upon differentiation (Figure 3C), we surmised that loss of *Yap1* would increase mitochondrial priming and thereby sensitize dESCs to activation of the apoptotic cascade.

**Figure 5.**
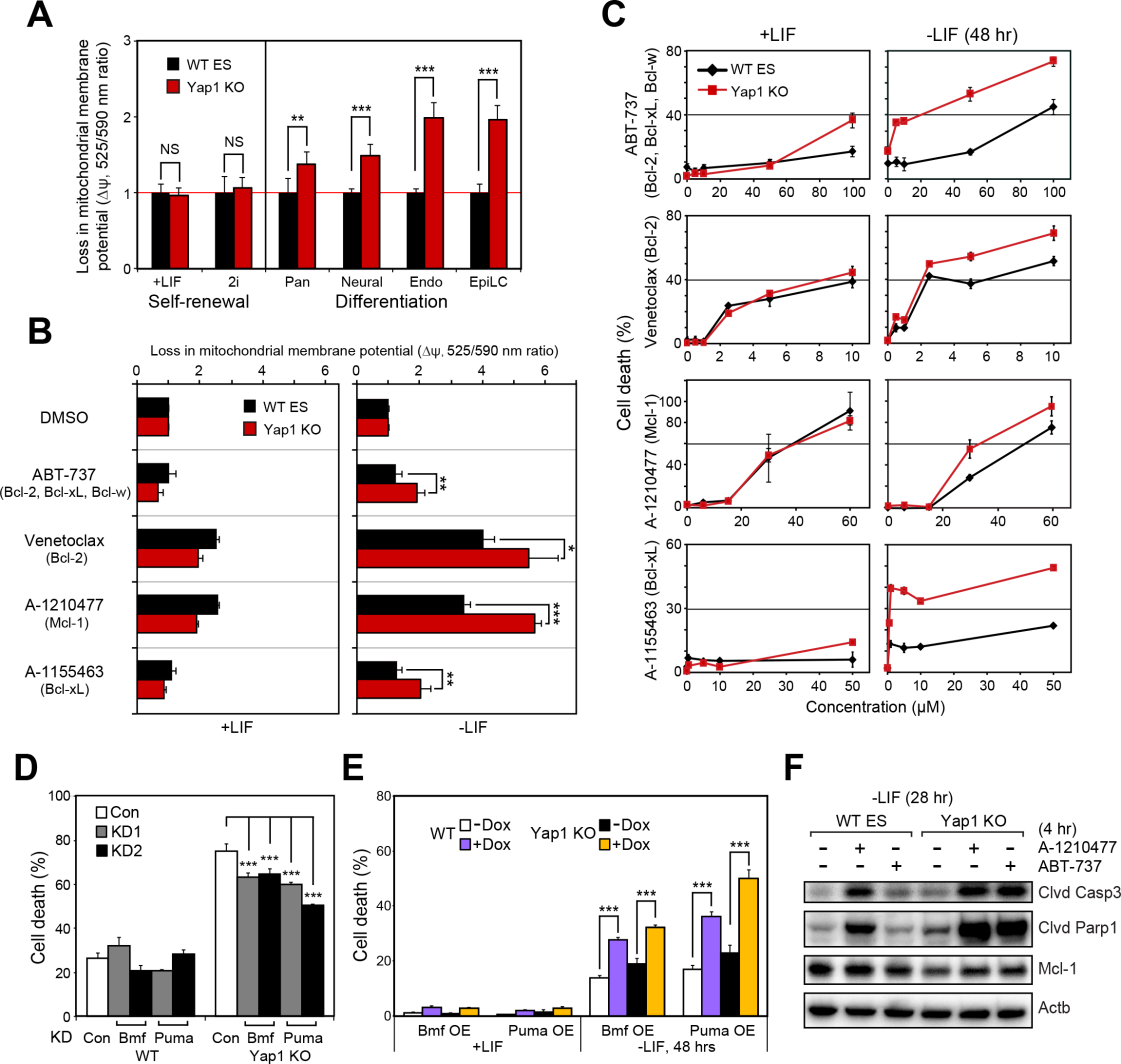
Yap1 regulates mitochondrial priming and addiction to anti-apoptotic proteins. (**A**) JC-10 mitochondrial membrane potential assay in WT and Yap1 KO cells during various forms of differentiation (72 hr for Pan and EpiLC, 48 hr for Neural and Endo) and self-renewal (maintained for an equal amount of time). Values (525/570 nm ratio, n = 6) corresponding to loss in ∆ψ (mitochondrial membrane potential) in Yap1 KO cells were normalized to WT cells. (**A**) JC-10 assay in WT and Yap1 KO cells in ±LIF after 12 hr of treatment with BH3 mimetics ABT-737, Venetoclax, A-1210477, and A1155463 (total differentiation time: 36 hr). Values (525/570 nm ratio) corresponding to loss in ∆ψ were normalized to DMSO as a control. (**C**) LDH assays of BH3 mimetic dose response curves after 24 hr of treatment in WT and Yap1 KO cells in ±LIF (48 hr differentiation). (**D**) LDH assay of WT and Yap1 KO cells after KD of Bmf or Puma in -LIF conditions (72 hr). (**E**) LDH assay of inducible Bmf and Puma OE (±Dox, 48 hr, 500 ng/mL) in WT and Yap1 KO cells in ±LIF (48 hr differentiation). (**F**) Immunoblot of cleaved Casp3, cleaved Parp1, and Mcl-1 in WT and Yap1 KO dESCs (28 hr) after 4 hr of treatment with BH3 mimetics A-1210477 (Mcl-1 inhibitor) and ABT-737 (inhibitor of Bcl-2, Bcl-xL, and Bcl-w). β-actin was used as a loading control. All data are expressed as mean ±standard deviation (n = 4 independent samples unless otherwise stated). Two sample two-tailed t-test compared to WT or whatever is specified on the y-axis: *=0.05 > P > 0.01. **=0.01 > P > 0.001. ***=0.001 ≥ P.

Using the JC-10 assay, we measured differences in mitochondrial priming between Yap1 KO and WT ESCs during differentiation, initially. Whereas mitochondria were equally primed during self-renewal, all four differentiation conditions (-LIF, neural, endoderm, EpiLC) resulted in a greater loss of mitochondrial membrane potential (Δψ) in Yap1 KO cells normalized to WT ESCs (Figure 5A). Next, we treated ESCs with a small panel of BH3 mimetics capable of inhibiting Bcl-2, Bcl-xL, Mcl-1, and/or Bcl-w to measure addiction to anti-apoptotic proteins. As expected, inhibition of anti-apoptotic proteins increased Δψ in dESCs more than in self-renewing ESCs (Figure 5B). Furthermore, deletion of *Yap1* significantly sensitized dESCs, but not undifferentiated ESCs, to Δψ loss post BH3 mimetic treatment. We then investigated whether the higher loss of Δψ in Yap1 KO cells correlated with greater rates of cell death. Our results showed that mere inhibition of anti-apoptotic proteins was sufficient to cause cell death, particularly in dESCs, even before apoptosis normally occurs during differentiation (Figure 5C). Strikingly, loss of *Yap1* significantly amplified cell death in response to BH3 mimetics at almost all concentrations tested, but only during differentiation (Figure 5C). Ablation of *Yap1* also enhanced addiction to Mcl-1 and Bcl-xL; inhibition of either protein resulted in 2-3x greater cell death in Yap1 KO than WT (Figure 5C). Thus, loss of *Yap1* leads to increased mitochondrial priming during differentiation, which subsequently sensitizes Yap1 KO to excessive activation of the apoptotic cascade. Importantly, despite how critical mitochondrial priming is to biomedical applications such as successful chemotherapy, almost no genes that regulate mitochondrial priming upstream of apoptosis-related proteins have been shown in any context.

**Figure 5—figure supplement 1.**
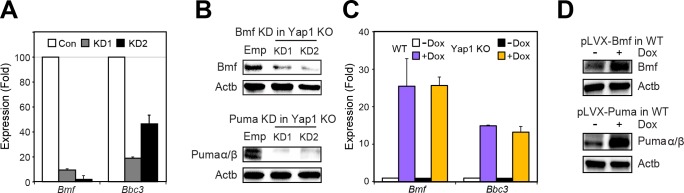
Verification of KD and OE of pro-apoptotic factors Bmf and Puma. (**A**) RT-qPCR measuring the expression of Bmf and Puma in Yap1 KO cells during differentiation (48 hr) relative to empty vector KD (n = 3). (**B**) Immunoblot of Bmf and Puma after KD in Yap1 KO cells during differentiation (48 hr) relative to empty vector KD. β-actin was used as a loading control. (**C**) RT-qPCR measuring the expression of Bmf and Puma in WT and Yap1 KO ESCs ± Dox (24 hr) in +LIF (n = 2). (**D**) Immunoblot of Bmf and Puma after OE in WT ESCs in +LIF. β-actin was used as a loading control.

### Manipulation of the levels of BH3-only proteins Bmf or Puma mildly affect rates of ESC death in the absence of Yap1 during differentiation

BH3 mimetics promote cell death by mimicking pro-apoptotic BH3-only proteins (Dai et al., 2016). In addition to lower expression of anti-apoptotic proteins, we observed higher expression of BH3-only genes in Yap1 KO cells relative to WT cells (Figure 3C). Though it is known that KD of Puma in self-renewing ESCs reduces sensitivity to cytotoxic agents (Huskey et al., 2015), roles of Bmf and Puma during differentiation are relatively unknown. Therefore, we performed KD of Bmf and Puma (Figure 5—figure supplement 1A and B) and this mildly reduced cell death in Yap1 KO cells during differentiation but not in WT ESCs (Figure 5D). Inducible OE of either factor (Figure 5—figure supplement 1C and D) accelerated apoptosis during differentiation, particularly in Yap1 KO cells, and Puma promoted cell death more strongly than Bmf (Figure 5E). This difference may be because Puma promiscuously binds to all known anti-apoptotic Bcl-2 family proteins, whereas Bmf binds only weakly to Mcl-1, preferring Bcl-2, Bcl-xL, and Bcl-w (Chen et al., 2005). Thus, although Yap1’s pro-survival function is primarily via activation of anti-apoptotic proteins, heightened expression of individual pro-apoptotic BH3-only proteins seems to contribute to enhanced cell death during differentiation of Yap1 KO cells. Finally, we used BH3 mimetics to probe differential roles of anti-apoptotic proteins during early differentiation. Mcl-1 expression was already reduced in Yap1 KO cells 28 hr after LIF withdrawal compared to WT ESCs. Yap1 KO cells were acutely sensitive to inhibition (4 hr) of either Mcl-1 or Bcl-2/Bcl-xL/Bcl-w, indicating increased mitochondrial priming even at such an early timepoint (Figure 5F). Since Mcl-1 is much more highly expressed than both Bcl-2 and Bcl-xL, we surmise that deficiency in its expression helps explain heightened apoptotic activation in Yap1 KO cells even before differences in Bcl-2 expression become apparent (Figure 3E).

### Modulation of anti-apoptotic proteins controls cell death during differentiation

**Figure 6.**
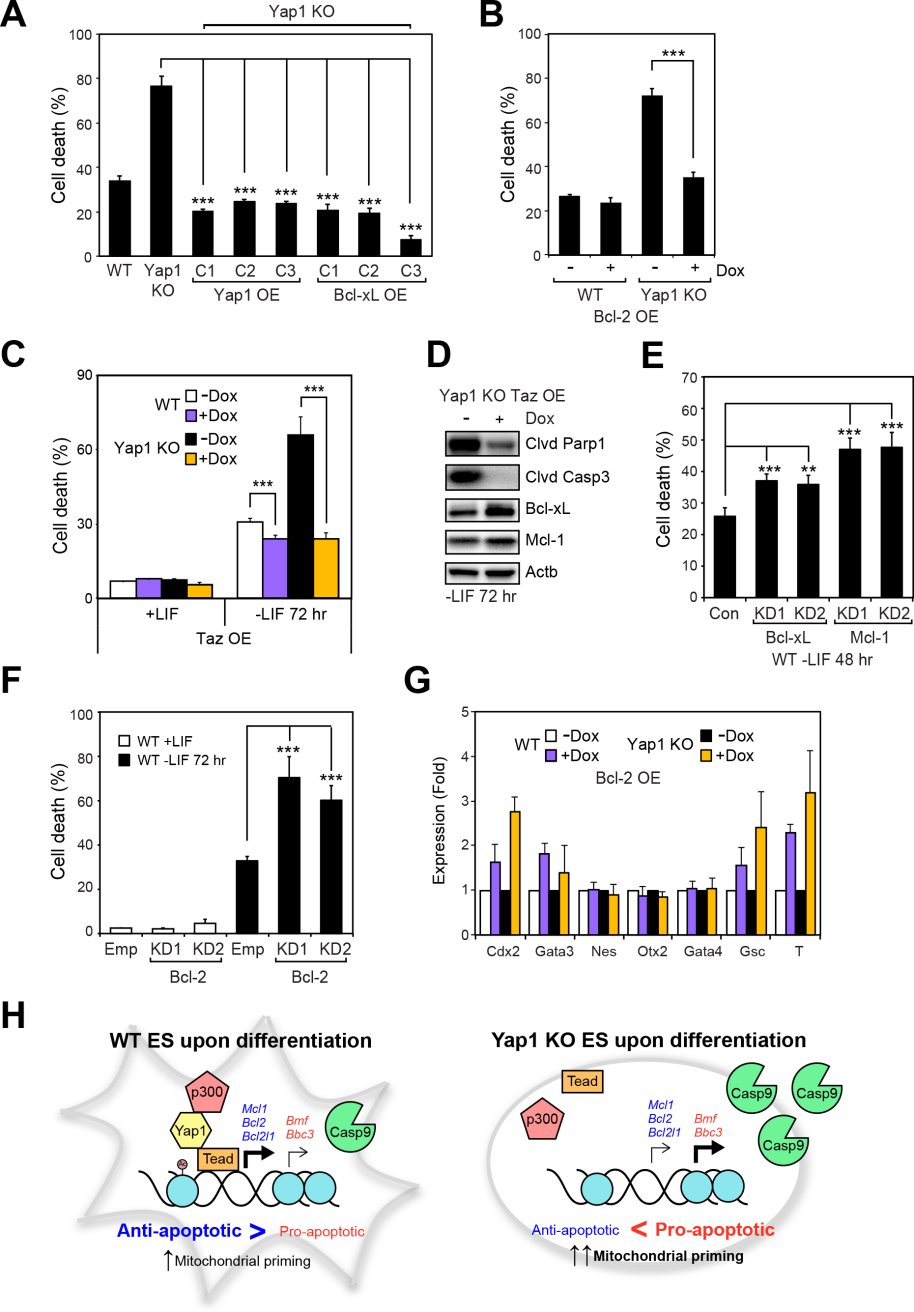
Overexpression of Taz or individual anti-apoptotic proteins fully rescues the survival defect in the absence of Yap1. (**A**) LDH assay of WT, Yap1 KO, and Yap1 KO constitutively overexpressing Bcl-xL or Yap1 in -LIF (72 hr). (**B**) LDH assay of inducible Bcl-2 (±Dox, 48 hr, 500 ng/mL) in WT and Yap1 KO cells -LIF (72 hr). (**C**) LDH assay of inducible Taz (±Dox, 48 hr, 500 ng/mL) in WT and Yap1 KO cells ± LIF (72 hr differentiation). (**D**) Immunoblot of cleaved Parp1, cleaved Casp3, Bcl-xL, and Mcl-1 in Yap1 KO cells inducibly overexpressing Taz (±Dox, 48 hr, 500 ng/mL) in -LIF (72 hr). (**E**) LDH assay of WT ESCs during differentiation (72 hr) after 48 hr KD of Bcl-xL or Mcl-1. (**F**) LDH assay of WT ESCs ± LIF (72 hr)±KD of Bcl-2. (**G**) RT-qPCR measuring the expression of lineage markers (trophectoderm: *Cdx2* and *Gata3*, ectoderm: *Nes* and *Otx2*, endoderm: *Gata4*, mesoderm: *Gsc* and *T*) in WT and Yap1 KO cells in -LIF (72 hr, n = 3). Expression is indicated as a fold change in +Dox samples relative to -Dox. (**H**) Model proposing roles for Yap1 specific to the exit from self-renewal. In complex with Tead factors like Tead4, Yap1 co-activates anti-apoptotic genes and mildly co-represses pro-apoptotic genes to dampen mitochondrial priming, which thus prevents hyperactivation of the apoptotic cascade through Casp9. All data are expressed as mean ±standard deviation (n = 4 independent samples unless otherwise stated). Two sample two-tailed t-test compared to WT or whatever is specified on the y-axis: *=0.05 > P > 0.01. **=0.01 > P > 0.001. ***=0.001 ≥ P.

Having shown that Yap1 directly regulates apoptosis-related genes and thus reduces mitochondrial priming during differentiation, we sought to characterize whether modulating individual Yap1 targets could control cell death during differentiation. We stably overexpressed Yap1 (as a positive control to complement the KO) and Bcl-xL (Figure 6—figure supplement 1A) and inducibly overexpressed Bcl-2 (Figure 6—figure supplement 1B, C and D) in Yap1 KO cells, which reduced cell death in Yap1 KO to levels comparable to WT (Figure 6A and B). Intriguingly, inducible OE of Taz, a Yap1 paralog also possessing a Tead-binding domain, reduced cell death in both Yap1 KO cells and WT cells to levels just below uninduced WT cells, perhaps via upregulation of Bcl-xL (Figure 6C and D), and Figure 6—figure supplement 1F). Conversely, KD of Bcl-xL, Mcl-1 (Figure 6—figure supplement 1F), or Bcl-2 (Figure 6—figure supplement 1G) in WT ESCs individually increased cell death during differentiation 1.5 to 2-fold compared to controls (Figure 6E and F, Figure 6—figure supplement 1H). Since Yap1 is crucial for ES differentiation, we questioned whether the apoptosis-related genes regulated by Yap1 might have some effect on differentiation efficiency. Surprisingly, we found that OE of Bcl-2 led to increased induction of trophectoderm (*Cdx2* and *Gata3*) and mesoderm markers (*Gsc* and *T*), while KD of Bcl-2 tended to reduce induction of lineage markers. However, KD of Bcl-xL or Mcl-1 had no effect on lineage marker induction (Figure 6G and Figure 6—figure supplement 1I and J). Taken together, these data clearly demonstrate that anti-apoptotic factors transcriptionally regulated by Yap1 are critical for dESC survival, and that OE or KD of each apoptotic factor can significantly shift the balance between survival and death. The results additionally suggest the previously unknown roles of Bcl-2 in regulation of ESC lineage specification, as only its roles in self-renewal have been deeply probed (Yamane et al., 2005). Our combined model of Yap1’s role in ESC differentiation is provided in Figure 6H.

**Figure 6—figure supplement 1.**
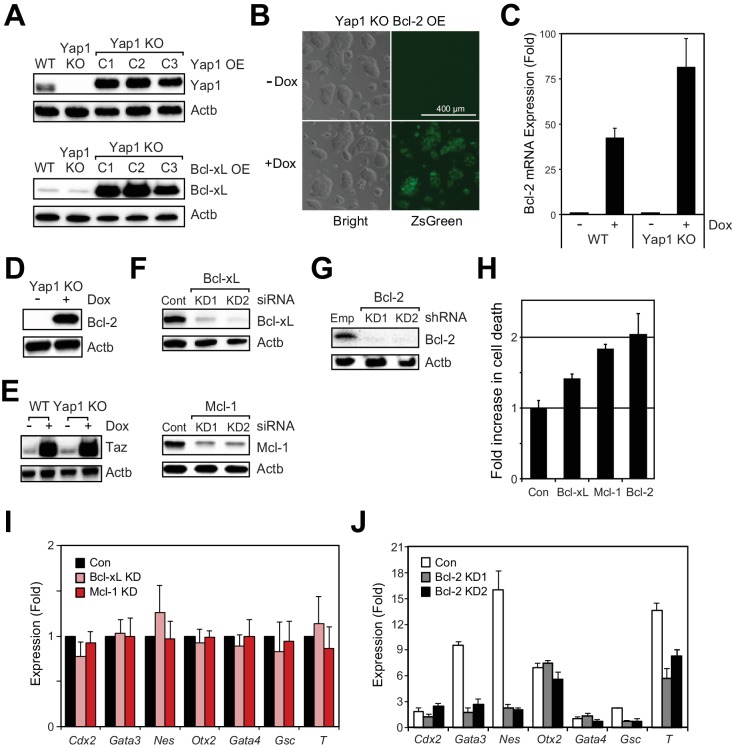
Modulation of the expression of individual anti- or pro-apoptotic genes influences cell death during differentiation. (**A**) Immunoblot of Yap1 and Bcl-xL after OE in Yap1 KO cells relative to WT or empty vector Yap1 KO in +LIF. β-actin was used as a loading control. (**B**) Representative brightfield and fluorescent microscopy images of Yap1 KO cells showing ZsGreen fluorescence ±Dox. Scale bar, 400 μm. (**C**) RT-qPCR measuring the expression of Bcl-2 in WT and Yap1 KO cells during differentiation (72 hr)±Dox (48 hr, 500 ng/mL). (**D**) Immunoblot of Bcl-2 in Yap1 KO cells ± Dox (48 hr, 500 ng/mL) in +LIF. (**E**) Immunoblot of Taz in WT and Yap1 KO cells ± Dox (48 hr, 500 ng/mL) in +LIF. (**F**) Immunoblot of Bcl-xL and Mcl-1 after siRNA KD in WT cells in -LIF (48 hr). (**G**) Immunoblot of Bcl-2 after shRNA KD in -LIF (72 hr). (**H**) Quantification of fold increase in cell death from Figure 6E and F observed upon KD of Bcl-xL, Mcl-1, or Bcl-2, relative to control KD, in -LIF (72 hr). (**I**) RT-qPCR measuring the expression of lineage markers in WT cells transfected with siRNA against Bcl-xL or Mcl-1 in -LIF (72 hr). Expression is indicated as a fold change compared to control siRNA. (**J**) RT-qPCR measuring the induction of lineage markers in WT cells transduced with shRNA against Bcl-2 in -LIF relative to +LIF (72 hr). All data are expressed as mean ±standard deviation (n = 3 independent samples). Two sample two-tailed t-test compared to WT or whatever is specified on the y-axis: *=0.05 > P > 0.01. **=0.01 > P > 0.001. ***=0.001 ≥ P.

## Discussion

Though ESCs experience 30% or more cell death during differentiation, regulators of this process remain largely unknown. In this study, we have shown that in the absence of *Yap1*, this proportion of cell death increases to 70–80%. Accordingly, we have demonstrated that Yap1 directly and strongly activates anti-apoptotic genes, in addition to mildly repressing pro-apoptotic genes, to promote survival during the stressful process of differentiation. Yap1 therefore attenuates the increase in mitochondrial priming during differentiation that threatens mitochondrial integrity and leads to Casp9 activation. OE of Yap1, its paralog Taz, or its anti-apoptotic targets in Yap1 KO cells reduces cell death to WT levels or even lower. Our proposed role for Yap1 as a pro-survival factor in ESCs is consistent with other studies done in cancer or epithelial contexts (Lin et al., 2015; Rosenbluh et al., 2012; Song et al., 2015; Zhao et al., 2016), but our work is the first study to show such a contextual, differentiation-specific role for Yap1 in ESCs.

Intriguingly, our Casp3 live imaging assay revealed that activation of Casp3 was extremely heterogeneous, with many cells changing their morphology during differentiation without detectable caspase activation. Future studies focusing on how individual cells make the molecular decision of differentiation vs. apoptosis will be desired. We hypothesize that relative changes in the expression of key pro- and anti-apoptotic genes at the single cell level, as well as lineage markers, shortly after LIF withdrawal could successfully predict whether an individual cell will differentiate or perish.

One unexpected finding from our study is that OE of Bcl-2 improved induction of essential trophectoderm and mesoderm markers, and KD of Bcl-2 (but not Mcl-1 or Bcl-xL) conversely hampered such induction. Elucidating the mechanism by which Bcl-2 accelerates induction of lineage markers is beyond the scope of this work but would enhance understanding of how apoptosis-related factors influence non-apoptotic processes such as differentiation. Additionally, we noted that Yap1 binding peaks on apoptosis-related genes are conserved across several human cancer cell types, which corroborates previous findings (Rosenbluh et al., 2012). Since addiction to anti-apoptotic factors is a defining characteristic of cancer cells, the regulation mechanisms we have elucidated may be broadly applicable to how Yap1 promotes tumorigenesis.

In sum, our study has clearly demonstrated that Yap1 robustly promotes survival of ESCs during differentiation by direct transcriptional regulation of apoptotic genes. Nearly all cells in the body originate from various progenitor cells, and since the process of differentiation is often fraught with error and stress, our research may spur advances in the regulation of the survival or death decision during cell fate changes in a broad variety of contexts.

## Materials and methods

**Key resources table:** 

Reagent type (species) or resource	Designation	Source or reference	Identifiers	Additional information
Antibody	Mouse anti -β-actin	Abgent	Cat#AM1829b RRID:AB_10664137	1:20,000 in 5% BSA
Antibody	Mouse anti-Yap1	Santa Cruz Biotechnology	Cat#sc-101199 RRID:AB_1131430	1:1000 in 5% milk
Antibody	Rabbit anti- cleaved caspase-3	Cell Signaling Technology	Cat#9661S RRID:AB_2341188	1:1000 in 5% BSA
Antibody	Mouse anti- cleaved Parp1	Cell Signaling Technology	Cat#9548S RRID:AB_2160592	1:1000 in 5% BSA
Antibody	Mouse anti- caspase-9	Cell Signaling Technology	Cat#9508S RRID:AB_10695598	1:1000 in 5% BSA
Antibody	Rabbit anti- Bcl-2	Cell Signaling Technology	Cat#3498S RRID:AB_1903907	1:1000 in 5% BSA (WB), 1:200 in 4% BSA, 1% NGS (ICC)
Antibody	Rabbit anti-Bcl-xL	Cell Signaling Technology	Cat#2764S RRID:AB_10695729	1:1000 in 5% BSA
Antibody	Rabbit anti-Mcl-1	Cell Signaling Technology	Cat#94296S RRID:AB_2722740	1:1000 in 5% BSA, 1:800 in 4% BSA, 1% NGS (ICC)
Antibody	Mouse anti-Puma	Santa Cruz Biotechnology	Cat#sc-374223 RRID:AB_10987708	1:500 in 5% BSA
Antibody	Rabbit anti-Bmf	Bioss	Cat#bs-7587R RRID: AB_2722741	1:1000 in 5% BSA
Antibody	Horse anti-mouse secondary, HRP- conjugated	Cell Signaling T echnology	Cat#7076P2 RRID: AB_330924	1:10,000 in TBST
Antibody	Goat anti-rabbit secondary, HRP- conjugated	Cell Signaling Technology	Cat#7074S RRID:AB_2099233	1:10,000 in TBST
Antibody	Goat anti-rabbit IgG Alexa Fluor 594	Thermo Scientific	Cat#R37117 RRID:AB_2556545	1:1000 in 4% BSA, 1% NGS (ICC)
Antibody	Dynabeads MyOne Streptavidin T1	Thermo Scientific	Cat#65601	1:2000 in 5% BSA
Antibody	Rabbit anti- Taz (Wwtr1)	Sigma Aldrich	HPA007415 RRID:AB_1080602	1:500 in 5% milk
Chemical compound, drug	Z-VAD-FMK	ApexBio	Cat#A1902	
Chemical compound, drug	Necrostatin-1	Selleck Chemicals	Cat#S8037	
Chemical compound, drug	CHIR99021	Selleck Chemicals	Cat#S2924	
Chemical compound, drug	PD184352	Selleck Chemicals	Cat#S1020	
Chemical compound, drug	IDE1	Cayman Chemical Company	Cat#13816	
Chemical compound, drug	Staurosporine	Cell Signaling Technology	Cat#9953S	
Chemical compound, drug	Polybrene	Millipore	Cat#TR‐ 1003‐G	
Chemical compound, drug	Puromycin	Thermo Scientific	Cat#A111 3803-02	
Chemical compound, drug	Geneticin /G418	Thermo Scientific	Cat#10131027	
Chemical compound, drug	ABT-737	Selleckchem	Cat#S1002	
Chemical compound, drug	Venetoclax /ABT-199	Selleckchem	Cat#S8048	
Chemical compound, drug	A-1210477	Selleckchem	Cat#S7790	
Chemical compound, drug	A-1155463	Selleckchem	Cat#S7800	
Chemical compound, drug	Lipofectamine 3000	Life Technologies	Cat#L3000008	
Chemical compound, drug	INTERFERin	Polyplus Transfection	Cat#409–10	
Chemical compound, drug	Verteporfin	Selleck Chemicals	S1786	
Chemical compound, drug	Recombinant Human /Mouse/Rat Activin A Protein	R and D Systems	338-AC-010	
Chemical compound, drug	Gibco FGF Basic Recombinant Mouse Protein	Thermo Fisher Scientific	PMG0034	
Chemical compound, drug	KnockOut Serum Replacement	Thermo Fisher Scientific	10828028	
Commercial assay or kit	Pierce LDH Cytotoxicity Assay Kit	Thermo Scientific	Cat#88954	
Commercial assay or kit	RNeasy Plus Mini Kit	Qiagen	Cat#74136	
Commercial assay or kit	qScript cDNA SuperMix	QuantaBio /VWR	Cat#101414–108	
Commercial assay or kit	PerfeCTa SYBR Green FastMix	VWR	Cat#95072–012	
Commercial assay or kit	Caspase-Glo 3/7	Promega	Cat#G8090	
Commercial assay or kit	Caspase-Glo 8	Promega	Cat#G8200	
Commercial assay or kit	Caspase-Glo 9	Promega	Cat#G8210	
Commercial assay or kit	Cell Meter JC-10 Mitochondrion Membrane Potential Assay Kit	AAT Bioquest	Cat#22800	
Commercial assay or kit	NEBNext Ultra II DNA Library Prep Kit for Illumina	New England Biolabs	Cat#E7645S	
Commercial assay or kit	Dual-Glo Luciferase Assay System	Promega	Cat#E2920	
Recombinant DNA reagent	pLKO-puro	Millipore Sigma	See table S3	
Recombinant DNA reagent	pLVX-TRE3G -ZsGreen1	Clontech	Cat#631164	
Recombinant DNA reagent	pCMV-Tet3G	Clontech	Cat#631164	
Recombinant DNA reagent	pCMV3- Bcl2l1/Bcl-xL	Sino Biological	Cat#MG50012-UT	
Recombinant DNA reagent	pGL3-promoter	Promega	Cat#E1761	
Recombinant DNA reagent	pRL-TK	Promega	Cat#E2231	
Recombinant DNA reagent	3149 pSFFV-neo Bcl-2 cDNA	AddGene	Cat#8750	
Recombinant DNA reagent	Mus musculus BCL2 binding component 3 (Bbc3), mRNA. NM_ 133234.2	GenScript	Cat#OMu19350D	
Recombinant DNA reagent	pcDNA3.1/ HisC-mTAZ	AddGene	Cat#31793	
Cell line (*Mus musculus*, male)	J1 Embryonic Stem Cells	ATCC	ATCC SCRC -1010	
Cell line (*M. musculus,* male)	CJ7 Embryonic Stem Cells	ENCODE	RRID:CVCL_C316	
Cell line (*M. musculus,* male)	ES-E14TG2a Embryonic Stem Cells	ATCC	ATCC CRL-1821	
Cell line (*Homo sapiens*)	HEK293T cells	ATCC	ATCC CRL-3216	
Software, algorithm	FlowJo	Treestar		
Software, algorithm	BoxPlotR	http://shiny.chemgrid.org/boxplotr/		
Software, algorithm	Java TreeView	http://jtreeview.sourceforge.net/		
Software, algorithm	AmiGO 2	http://amigo.geneontology.org		
Software, algorithm	Primer3	http://primer3.ut.ee/		
Software, algorithm	HOMER	http://homer.ucsd.edu/homer/		
Software, algorithm	GOrilla	http://cbl-gorilla.cs.technion.ac.il/		
Software, algorithm	Cistrome	http://cistrome.org/db/#/		
Software, algorithm	Galaxy	http://cistrome.org/ap/		
Software, algorithm	SRA Toolkit	https://trace.ncbi.nlm.nih.gov/Traces/sra/sra.cgi?view=toolkit_doc		
Software, algorithm	Bowtie 2	http://bowtie-bio.sourceforge.net/bowtie2/index.shtml		
Software, algorithm	MACS2	https://github.com/taoliu/MACS		
Software, algorithm	Vassar Stats	http://vassarstats.net/matrix2.html		
Software, algorithm	Integrated Genome Viewer	http://software.broadinstitute.org/software/igv/		
Software, algorithm	ZEN Microscope Software	https://www.zeiss.com/microscopy/int/downloads/zen.html		
Software, algorithm	ImageJ	https://imagej.nih.gov/ij/index.html		
Software, algorithm	STAR	https://github.com/alexdobin/STAR		

### Cell culture

J1, CJ7, and E14TG2a (E14) male mouse ESCs were cultured on 0.1% gelatin-coated plates in Dulbecco’s Modified Eagle’s Medium (DMEM, Gibco) supplemented with 18% fetal bovine serum (BioWest), MEM nonessential amino acids (Gibco), EmbryoMax nucleosides (Millipore), 50 U/mL penicillin/streptomycin/L-glutamine (PSG, Gibco), 100 µM β-mercaptoethanol, and 1000 U/mL recombinant mouse leukemia inhibitory factor (LIF, Millipore) in a 37°C with 5% CO_2_. Media was changed daily, and cells were passaged every 2 days. J1, CJ7, E14TG2a mouse ES cells and HEK293T cells were all obtained from ATCC (except CJ7 line was obtained from Dr. Stuart Orkin), confirmed by partial genomic DNA sequencing. Mycoplasma contamination was not detected by PCR based methods.

### Cell death assay

ESCs were seeded at a density of 2 × 10^5^ cells/mL in the indicated media type in a clear 96-well plate. Inhibitors were administered 24 hr after seeding at a concentration of 50 μM (Z-VAD-FMK, ApexBio; Necrostatin-1, Selleckchem). At the indicated timepoints, lactate dehydrogenase (LDH) activity was quantified in the supernatant using the Pierce LDH Cytotoxicity Assay Kit (Thermo Scientific) according to the manufacturer’s instructions. A_680nm_ values were first subtracted as background noise. Then, absorbance from an average of 3 media-only wells (reflecting background LDH activity) was subtracted from every sample’s A_490nm_ value. Data were normalized to wells that had been lysed completely using the provided lysis buffer to establish a benchmark for 100% cell death.

### Cell differentiation

For nonspecific differentiation, ESCs were washed in -LIF medium and then seeded at the cell densities specified below in each assay, as well as passaged on day one after seeding. Cells were assayed at 24, 48, 60, and/or 72 hr according to the experiment. For neural differentiation, ESC were first maintained in NDiff 227 medium (N2B27, Clontech) supplemented with 3 μM CHIR99021 (Selleck Chemicals) and 1 μM PD184352 (Selleck Chemicals), defined as 2i, to promote self-renewal in the absence of serum and LIF. Differentiation occurred in N2B27 in the absence of 2i and assayed at 24, 48, and 72 hr. For definitive endoderm differentiation, ESCs were grown in DMEM supplemented with 1% FBS, PSG, and 5 μM IDE1 (Cayman Chemical Company) and assayed at 48 hr. For EpiLC differentiation, ESCs were grown in N2B27 supplemented with 20 ng/mL activin A (R and D Systems), 12 ng/mL bFGF (Thermo Fisher), and 1% KOSR (Thermo Fisher) as previously shown (Hayashi et al., 2011), and assayed at 72 hr. For verteporfin-related experiments, verteporfin was diluted in DMSO to a concentration of 1 mM and then further diluted in fresh media during media changes, and cells were protected from light with aluminum foil. HEK293T cells (ATCC CRL-3216) were cultured in DMEM supplemented with 10% FBS and PSG. All cells were grown at 37°C in the presence of 5% CO_2_.

### Flow cytometry

For analysis of externalized phosphatidylserine and active caspase-3, ESCs were seeded at a density of 4 × 10^5^ cells per well on a six well plate in medium with or without LIF. After incubating cells for the indicated number of hours, cells were gently detached and dissociated into a single cell suspension using Accutase (Biolegend). Cells were then resuspended in 200 μL 1X Annexin V binding buffer plus 5 μL of 0.2 mM NucView 488 caspase-3 substrate solution and 5 μL CF 594 annexin V solution (Biotium). Cells were incubated in the dark at room temperature for 30 mins, then centrifuged (1000 rpm, five mins) at 4°C and washed. Then, stained cells were filtered using a 70 μm cell strainer to remove clumps (Celltreat). Flow cytometry was performed on a BD LSRFortessa SORP Flow Cytometer (BD Biosciences) and analysis was carried out with FlowJo (Treestar).

### Immunoblotting

For generating lysates suitable for Western blot, ESCs were cultivated under various conditions (e.g., differentiation) in 6-well, 12-well, or 24-well gelatin-coated plates. After various conditions were met cells were quantified using 0.2% trypan blue to distinguish viable from nonviable cells. Then, up to 4 × 10^6^ cells were directly lysed via addition of 2x Laemmli Sample Buffer (Bio-Rad) supplemented with 5% β-mercaptoethanol (Millipore Sigma). Lysates were heated at 95°C for five mins, then cooled to room temperature and routinely stored at −20°C. Lysates were loaded into gels such that either the absolute number of viable cells (quantified by trypan blue) or amount of protein (quantified by the Pierce BCA Assay, Thermo Scientific) loaded in each well was the same. Up to 15 μL of lysate was run on a 4–20% Mini-PROTEAN TGX Stain-Free protein gel (Bio-Rad) or a 10% TGX FastCast gel (Bio-Rad) in denaturing conditions at 130V for 50–70 mins followed by semi-dry transfer using the Trans-Blot Turbo Transfer System (Bio-Rad) onto 0.2 µm nitrocellulose or methanol-activated PVDF membranes (Bio-Rad). Successful protein transfer was verified with Ponceau S staining (Amresco) or stain-free fluorescent crosslinking.

After blocking with 5% bovine serum albumin (BSA) or 5% skim milk (for Yap1 and β-actin only) in Tris-buffered saline containing 0.1% Tween 20 (TBST), membranes were incubated overnight with primary antibodies, diluted in 5% BSA as described in the paragraph below. The following day, membranes were washed, incubated with secondary antibodies, washed again, incubated with Amersham ECL Prime Western Blotting Detection Reagent (GE Healthcare) and visualized on a ChemiDoc XRS+ (Bio-Rad). β-actin was used as a loading control.

Primary antibodies (purchased from Cell Signaling Technology unless otherwise specified) along with dilutions used were the following: β-actin (Abgent #AM1829b, 1:20,000), Yap1 (Santa Cruz Biotechnology #sc-101199, 1:1000), Casp8 (#4927S, 1:1000), Casp3 (#9662S, 1:1000), Cleaved Caspase-3 (#9661S, 1:1000), Cleaved Parp1 (#9548S, 1:1000), Caspase-9 (#9508S, 1:1000), Bcl-2 (#3498S, 1:1000), Bcl-xL (#2764S, 1:1000), Mcl-1 (#94296S, 1:1000), Tead4 (Abcam #ab58310, 1:5000), Puma (Santa Cruz Biotechnology #sc-374223, 1:500), and Bmf (Bioss #bs-7587R, 1:1000). HRP-conjugated secondary antibodies (purchased from Cell Signaling Technology), used at a dilution of 1:10,000 in TBST, were horse anti-mouse (#7076P2) and goat anti-rabbit (#7074S).

### Lentiviral production and infection and transposon-mediated gene integration

Lentiviruses were used to transduce shRNA and overexpression constructs. Bacterial glycerol stocks containing the appropriate shRNA were purchased from Millipore Sigma. A complete list of shRNA can be found in supplemental table S3. HEK293T cells were seeded at a density of 1.2 × 10^6^ cells per well on a six well plate. After reaching a confluency of 50–60%, cells were transfected with 1.2 μg of an shRNA-containing pLKO-puro vector (Millipore Sigma) as well as 800 ng pCMV‐Δ8.9 and 400 ng VSVG packaging plasmids with Fugene 6 (Promega) using the manufacturer’s protocol. For inducible overexpression (OE), a pLVX-IRES-ZsGreen1 vector (Clontech) containing the gene of interest and pLVX-TRE3G vector (Clontech) were transfected separately with packaging plasmids. After 18 hr of overnight incubation, HEK293T medium was replaced with ES medium. Then, two days after transfection, medium was supplemented with HEPES to a final concentration of 15 mM to act as an additional buffer, and the supernatant (which contains lentiviral particles) was filtered through a 0.45‐μm Supor membrane (PALL). ESCs were infected at a density of 2 × 10^5^ cells/mL in medium supplemented with 10 μg/mL polybrene (Millipore). 48 hr post-infection, ESCs were selected with puromycin (Thermo Scientific) or geneticin/G418 (Thermo Scientific). Given that cells were passaged every two days, relevant experiments were performed within five passages of the initial infection.

As an alternative method for inducible OE, for the *Wwtr1* gene (Taz), a pSBtet-GP vector (AddGene) with luciferase replaced by a multiple cloning site and cloned with the gene of interest. The resultant construct was transfected along with a transposase-containing pCMV(CAT)T7-SB100 vector (AddGene) into ESCs at a density of 6 × 10^5^ cells/mL. Selection with puromycin occurred 24 hr later. All relevant experiments were performed within five passages of the initial transfection. Doxycycline (Fisher Scientific) was used at a concentration of 500 ng/mL for all inducible OE experiments. cDNAs for all OE experiments were obtained from either vectors (Bcl-xL - Sino Biological, Bcl-2–3149 pSFFV-neo Bcl-2 cDNA from AddGene, Puma - GenScript, Taz - pcDNA3.1/HisC-mTAZ from AddGene) or full-length mouse ESC cDNA reverse transcribed using the ProtoScript II First Strand cDNA Synthesis Kit from New England Biolabs (Bmf). All inserts were confirmed by Sanger sequencing.

### Caspase activity assay

For determination of caspase activity, the Caspase-Glo 3/7, 8, and 9 Assay Systems (Promega) was used. ESCs were seeded at a density of 1 × 10^5^ cells/mL in a white-walled 96-well plate (Millipore Sigma). At the indicated timepoints, cells were assayed using the respective kits according to manufacturer’s instructions. After subtracting the noise from blank wells (containing media but no cells), luminescent signals in each well were normalized to the cell number.

### Gene expression analysis

RNA-seq data was downloaded from Gene Expression Omnibus. Yap1 KD data belonged to the series GSE69669. Samples corresponding to accession numbers GSM1706496, GSM1706495, GSM1706489, and GSM1706488 were used for analysis. Boxplots were generated using BoxPlotR (http://shiny.chemgrid.org/boxplotr/) where whiskers extend to the 5^th^ and 95^th^ percentiles. Gene lists were taken from AmiGO 2 (http://amigo.geneontology.org), specifically positive (GO:2001244) and negative (GO:2001243) regulation of intrinsic apoptotic signaling pathway. Lists were double-checked for any genes known to behave differently than annotated in the ES cell context. Genes that were not expressed were removed from the analysis to reduce noise. Gene ontology analysis was carried out using GOrilla (http://cbl-gorilla.cs.technion.ac.il/) using the ‘two unranked lists of genes’ option, where the background list was populated by all genes listed in the RNA-seq output.

For RT-qPCR, total RNA was extracted from cells with the RNeasy Plus Mini Kit (Qiagen). Then, 600 ng of RNA was reverse transcribed into cDNA using the qScript cDNA SuperMix from QuantaBio (VWR). Next, qPCR was performed in 20 μL reactions using the PerfeCTa SYBR Green FastMix (VWR) plus 6 ng of cDNA and 250 nM forward and reverse primers. Primers were designed using Primer3 (http://primer3.ut.ee/) such that each primer amplified the junction between two or more exons, and their specificity as well as lack of primer dimer formation was verified with melt curve analysis showing one peak. Relative expression was normalized to Gapdh using the 2^-ΔΔCT^ method. All reactions were performed at least in triplicate on a StepOnePlus Real-Time PCR System (Applied Biosystems). All primer sequences are listed in Supplemental Table S1.

### Immunofluorescence

ESCs were seeded (6 × 10^5^ cells/mL) on a gelatin-coated µ-Slide VI 0.4 (Ibidi). For cells growing in -LIF conditions, cells were differentiated on a 10 cm plate for one day before seeding the µ-Slide and seeded at 2 × 10^5^ cells/mL. After an additional 2 days, cells were thoroughly washed with Dulbecco’s phosphate-buffered saline (DPBS) and fixed using 4% paraformaldehyde (freshly cracked with 70 mM NaOH at 70°C) for 15 mins. For mitochondrial staining, after washing but before fixation, cells were incubated in 300 nM MitoTracker Deep Red FM in OptiMem for 30 mins. Cells were washed again with DPBS and then permeabilized with 0.3% Triton X-100 in PBS for five mins. After washing once more, samples were blocked using IF blocking solution (4% BSA and 1% normal goat serum diluted in DPBS) for one hour, then incubated overnight with Bcl-2 (1:200) or Mcl1 (1:800) primary antibodies diluted in IF blocking solution. Then, samples were washed thoroughly followed by incubation with fluorescent secondary antibody (goat anti-rabbit IgG Alexa Fluor 594 from Thermo Scientific) diluted 1:1000 for one hour. After further washing, ProLong Glass Antifade Mountant with NucBlue (Thermo Scientific) was added to the samples, which were allowed to cure for 18–24 hr at room temperature. Slides were then imaged using a Zeiss LSM 710 Confocal Microscope using the Plan-Apo 63X (oil) objective and images were processed using ZEN microscope software. Colocalization was quantified using Zen software by setting the crosshairs such that noise was restricted to the lower left quadrant, and the same crosshair coordinates were used for all samples. Intensity was quantified using ImageJ and normalized to the number of discrete nuclei (stained by NucBlue) that could reasonably be assigned to separate cells.

### Chromatin immunoprecipitation followed by NextGen sequencing (ChIP-seq)

After reaching ~80% confluency in a 15 cm plate, BirA ESCs with or without FLAG-Bio-Yap1 were crosslinked with 1% formaldehyde for 7 min at room temperature and constant shaking. Formaldehyde was quenched with addition of glycine to a final concentration of 125 mM along with shaking for 5 min. Cells were then sonicated using a Bioruptor (Diagenode), and sheared chromatin including DNA fragments ~ 300 bp in length were used for immunoprecipitation with Dynabeads MyOne Streptavidin T1 (Thermo Scientific). Sequencing libraries were prepared with the enriched ChIP sample using the NEBNext Ultra II DNA Library Prep Kit for Illumina (New England Biolabs) and sequenced using the Illumina HiSeq 4000 at the UT Austin Genomic Sequencing and Analysis Facility (GSAF).

### ChIP-seq data analysis

Public ChIP-seq data sets were downloaded from Cistrome (http://cistrome.org/db/#/). When possible, only data sets that passed all of Cistrome’s quality control conditions were used. To determine pairwise correlations between ChIP-seq data sets, human YAP1 ChIP-seq peaks.bed files were sent directly to Galaxy (http://cistrome.org/ap/) and peaks were assigned to genes using the BETA-minus functionality (assembly hg19). For mouse Tead factor ChIP-seq datasets and our own Yap1 ChIP-seq data, fastq files were directly processed using the SRA Toolkit, and 75 bp reads were mapped onto the mouse genome (assembly mm9) using Bowtie 2. Peaks were then called using model-based analysis of ChIP-seq (MACS2). For comparison with p300 ChIP-seq data, Bowtie two output was used to compare target overlap within a window of 6 kb of the Yap1 peak center (in dESCs) using a bin size of 100 bp. The apoptosis gene list was retrieved from AmiGO 2 (GO:0006915). Binding scores for all genes were then used for pairwise correlations using Vassar Stats (http://vassarstats.net/matrix2.html) and correlations were visualized using Java TreeView (http://jtreeview.sourceforge.net/). Signal tracks were visualized using Integrated Genome Viewer (IGV, http://software.broadinstitute.org/software/igv/).

For our own Yap1 ChIP-seq data, motif analysis was performed using HOMER (http://homer.ucsd.edu/homer/motif/fasta.html). Peak to gene features were assigned using in-house Perl code. Binding sites were assigned to genomic features according to the following hierarchy: promoter (±2 kb of the TSS)>upstream (2–20 kb upstream of the TSS)>intron > exon>intergenic (all other binding sites that did not fit the other categories). Gene ontology (GO) analysis was performed using GOrilla (http://cbl-gorilla.cs.technion.ac.il/) using the two unranked lists of genes (target and background lists) setting. For the target list, all the genes with a peak score (normalized to BirA) greater than two were included. These Yap1 target genes were further sorted into either upregulated upon Yap1 KD (log2(KD/control)≥0.5) or downregulated ((log2(KD/control)≤−0.5). For the background list, all the genes from the bed file (20,422) were included. The top five GO terms (relative to -log10(p-value)), plus the top apoptosis-related GO term, were then graphed.

### Dual luciferase reporter assay

ESCs were seeded (6 × 10^5^ cells/mL when comparing Yap1 OE to empty, 4 × 10^5^ cells/mL when comparing Yap1 KO to WT) in a white-walled 96-well plate. Cells were transfected with 40 ng pGL3-promoter (Promega) containing firefly luciferase downstream of the SV40 promoter plus putative Yap1-responsive regulatory elements cloned from genomic mouse DNA. Simultaneously, as an internal control, cells were co-transfected with 40 ng pRL-TK containing Renilla luciferase downstream of the HSV-thymidine kinase promoter. During Yap1 OE experiments, half of the wells were transfected with 40 ng of a FLAG-Bio vector containing either Yap1, mutant Yap1 (Ser79Ala) generated by site-directed mutagenesis via the NEBuilder HiFi DNA Assembly Kit (New England Biolabs), or no insert downstream of the EF-1α promoter. All transfections related to luciferase were performed with Lipofectamine 3000 (Life Technologies). Cells were incubated for 16 hr before changing the media, and luciferase activity was measured by the Dual-Glo Luciferase Assay a total of 24 hr after transfection System (Promega). Firefly luciferase signal was normalized to Renilla luciferase signal, and then the signal of each regulatory element-containing construct was normalized to pGL3-promoter. All regulatory element sequences tested are listed in Supplemental Table S2. The NEBuilder HiFi DNA Assembly Kit was used to assemble the Bcl-2 tandem enhancer as well as the Mcl-1 distal enhancer with Tead site deletion (using overlapping homology excluding the Tead binding motif).

### Mitochondrial priming and loss of membrane potential

Mitochondrial membrane potential loss (∆ψ) was measured as a change in the 525/570 nm ratio relative to the DMSO-treated control using the Cell Meter JC-10 Mitochondrion Membrane Potential Assay Kit (AAT Bioquest) according to the manufacturer’s instructions after 12 hr of incubation with either BH3 mimetic or various timepoints of differentiation (72 hr for -LIF and EpiLC, 48 hr for neural ectoderm and endoderm). BH3 mimetics ABT-737 (Oltersdorf et al., 2005), Venetoclax/ABT-199 (Souers et al., 2013), A-1210477 (Leverson et al., 2015), and A-1155463 (Tao et al., 2014) were applied to ESCs or dESCs (after 24 hr of differentiation) at the concentrations indicated in the figure. Cell death was measured using the LDH assay as described above after 24 hr of incubation with the BH3 mimetic (48 hr total after LIF withdrawal).

### siRNA knockdown

MISSION siRNA was purchased from Millipore Sigma. Duplexes targeting *Mcl1* (NM_008562: SASI_Mm01_00048593, SASI_Mm02_00314161, SASI_Mm01_00048594) as well as *Bcl2l1* (NM_009743: SASI_Mm02_00316924, SASI_Mm02_00316925, SASI_Mm02_00316926) were ordered and resuspended at a concentration of 25 μM in 5X siRNA buffer (Dharmacon) diluted to 1X RNase-free water (Thermo Scientific). siRNA was reverse transfected into ESCs (6 × 10^5^ cells/mL) at a final concentration of 75 nM using INTERFERin according to the manufacturer’s protocol (Polyplus Transfection). MISSION siRNA Fluorescent Universal Negative Control #1 conjugated to 6-FAM was used as both a transfection control and as a non-targeting siRNA control. After verifying KD at the protein level by Western blot, the best two siRNAs were chosen for further experiments. All shRNA and siRNA TRC/ID numbers, and shRNA sequences (or the target position where siRNA is predicted to bind) are listed in supplemental table S3.

### Data, software, and code availability

Yap1 ChIP-seq data generated in this study has been uploaded to Gene Expression Omnibus under accession number GSE112606. Code used to analyze raw sequencing files using the programs STAR, Bowtie2, MACS, and Homer is available in the code file included with this manuscript (Source code file 1).

## Additional files

Source code 1. Code used to analyze raw sequencing files using the programs STAR, Bowtie2, MACS, and Homer.

Supplementary file 1. Supplementary Table S1. Table of RT-qPCR primers used for qPCR gene expression assays in this study. Primers were designed using Primer3 and verified by melt curve analysis. Supplementary Table S2. Table of cloning primers used for dual luciferase assay including chromosome coordinates (using mm9) and regulatory element length. Supplementary Table S3. Table of shRNA and siRNA used in KD experiments including target, ID, and sequence or target position.

Transparent reporting form

### Data availability

Sequencing data have been deposited in GEO under accession code GSE112606.

The following dataset was generated:

Bum-KyuLeeLucyLeBlancJonghwanKim2018Yap1 safeguards mouse embryonic stem cells from excessive apoptosis during differentiationNCBI Gene Expression OmnibusGSE11260610.7554/eLife.40167PMC630785930561326

The following previously published datasets were used:

DiepenbruckMWaldmeierLIvanekRBerningerPArnoldPvanNimwegen EChristoforiG2014Tead2 expression levels control the subcellular distribution of Yap and Taz, zyxin expression and epithelial-mesenchymal transition.NCBI Gene Expression OmnibusGSE5570910.1242/jcs.13986524554433

ZanconatoFForcatoMBattilanaGAzzolinLQuarantaEBodegaBRosatoABicciatoSCordenonsiMPiccoloS2015Genome-wide association between YAP/TAZ/TEAD and AP-1 at enhancers drives oncogenic growth.NCBI Gene Expression OmnibusGSE6608110.1038/ncb3216PMC618641726258633

SteinCBardetAFRomaGBerglingSClayIRuchtiAAgarinisCSchmelzleTBouwmeesterTSchübelerDBauerA2015YAP1 Exerts Its Transcriptional Control via TEAD-Mediated Activation of Enhancers.NCBI Gene Expression OmnibusGSE6185210.1371/journal.pgen.1005465PMC454660426295846

ChungHLeeBKUpretyNShenWLeeJKimJ2016Yap1 is dispensable for self-renewal but required for proper differentiation of mouse embryonic stem (ES) cellsNCBI Gene Expression OmnibusGSE6966910.15252/embr.201540933PMC481877026917425

HuYZhangZKashiwagiMYoshidaTJoshiIJenaNSomasundaramREmmanuelAOSigvardssonMFitamantJEl-BardeesyNGounariFVanEtten RAGeorgopoulosK2016Superenhancer reprogramming drives a B-cell-epithelial transition and high-risk leukemia.NCBI Gene Expression OmnibusGSE8689710.1101/gad.283762.116PMC506624027664237

ObierNCauchyPAssiSAGilmourJLie-A-LingMLichtingerMHoogenkampMNoaillesLCockerillPNLacaudGKouskoffVBoniferC2016Cooperative binding of AP-1 and TEAD4 modulates the balance between vascular smooth muscle and hemogenic cell fate.NCBI Gene Expression OmnibusGSE7932010.1242/dev.139857PMC520104527802171

## References

[bib1] Bao Q, Shi Y (2007). Apoptosome: a platform for the activation of initiator caspases. Cell Death and Differentiation.

[bib2] Bashamboo A, Taylor AH, Samuel K, Panthier JJ, Whetton AD, Forrester LM (2006). The survival of differentiating embryonic stem cells is dependent on the SCF-KIT pathway. Journal of Cell Science.

[bib3] Belmokhtar CA, Hillion J, Ségal-Bendirdjian E (2001). Staurosporine induces apoptosis through both caspase-dependent and caspase-independent mechanisms. Oncogene.

[bib4] Borowiak M, Maehr R, Chen S, Chen AE, Tang W, Fox JL, Schreiber SL, Melton DA (2009). Small molecules efficiently direct endodermal differentiation of mouse and human embryonic stem cells. Cell Stem Cell.

[bib5] Brodowska K, Al-Moujahed A, Marmalidou A, Meyer Zu Horste M, Cichy J, Miller JW, Gragoudas E, Vavvas DG (2014). The clinically used photosensitizer verteporfin (VP) inhibits YAP-TEAD and human retinoblastoma cell growth in vitro without light activation. Experimental Eye Research.

[bib6] Buecker C, Srinivasan R, Wu Z, Calo E, Acampora D, Faial T, Simeone A, Tan M, Swigut T, Wysocka J (2014). Reorganization of enhancer patterns in transition from naive to primed pluripotency. Cell Stem Cell.

[bib7] Chen L, Willis SN, Wei A, Smith BJ, Fletcher JI, Hinds MG, Colman PM, Day CL, Adams JM, Huang DC (2005). Differential targeting of prosurvival Bcl-2 proteins by their BH3-only ligands allows complementary apoptotic function. Molecular Cell.

[bib8] Chen L, Chan SW, Zhang X, Walsh M, Lim CJ, Hong W, Song H (2010). Structural basis of YAP recognition by TEAD4 in the hippo pathway. Genes & development.

[bib9] Chung H, Lee BK, Uprety N, Shen W, Lee J, Kim J (2016). Yap1 is dispensable for self-renewal but required for proper differentiation of mouse embryonic stem (ES) cells. EMBO Reports.

[bib10] Czabotar PE, Westphal D, Dewson G, Ma S, Hockings C, Fairlie WD, Lee EF, Yao S, Robin AY, Smith BJ, Huang DC, Kluck RM, Adams JM, Colman PM (2013). Bax crystal structures reveal how BH3 domains activate Bax and nucleate its oligomerization to induce apoptosis. Cell.

[bib11] Dai H, Meng XW, Kaufmann SH (2016). Mitochondrial apoptosis and BH3 mimetics. F1000Research.

[bib12] Deng J (2017). How to unleash mitochondrial apoptotic blockades to kill cancers?. Acta Pharmaceutica Sinica B.

[bib13] Dravid G, Ye Z, Hammond H, Chen G, Pyle A, Donovan P, Yu X, Cheng L (2005). Defining the role of Wnt/beta-catenin signaling in the survival, proliferation, and self-renewal of human embryonic stem cells. Stem Cells.

[bib14] Duval D, Reinhardt B, Kedinger C, Boeuf H (2000). Role of suppressors of cytokine signaling (Socs) in leukemia inhibitory factor (LIF) -dependent embryonic stem cell survival. The FASEB Journal.

[bib15] Ehmer U, Sage J (2016). Control of proliferation and cancer growth by the hippo signaling pathway. Molecular Cancer Research.

[bib16] Fischer U, Jänicke RU, Schulze-Osthoff K (2003). Many cuts to ruin: a comprehensive update of caspase substrates. Cell Death & Differentiation.

[bib17] Francelin CVL (2011). Apoptosis and the developing T cells. Journal of Clinical & Cellular Immunology.

[bib18] Fuchs Y, Steller H (2011). Programmed cell death in animal development and disease. Cell.

[bib19] Hansen CG, Moroishi T, Guan KL (2015). YAP and TAZ: a nexus for Hippo signaling and beyond. Trends in Cell Biology.

[bib20] Hao Y, Chun A, Cheung K, Rashidi B, Yang X (2008). Tumor suppressor LATS1 is a negative regulator of oncogene YAP. The Journal of Biological Chemistry.

[bib21] Hayashi K, Ohta H, Kurimoto K, Aramaki S, Saitou M (2011). Reconstitution of the mouse germ cell specification pathway in culture by pluripotent stem cells. Cell.

[bib22] Hu Q, Wu D, Chen W, Yan Z, Shi Y (2013). Proteolytic processing of the caspase-9 zymogen is required for apoptosome-mediated activation of caspase-9. Journal of Biological Chemistry.

[bib23] Huang J, Wu S, Barrera J, Matthews K, Pan D (2005). The hippo signaling pathway coordinately regulates cell proliferation and apoptosis by inactivating yorkie, the drosophila homolog of YAP. Cell.

[bib24] Huskey NE, Guo T, Evason KJ, Momcilovic O, Pardo D, Creasman KJ, Judson RL, Blelloch R, Oakes SA, Hebrok M, Goga A (2015). CDK1 inhibition targets the p53-NOXA-MCL1 axis, selectively kills embryonic stem cells, and prevents teratoma formation. Stem Cell Reports.

[bib25] Jaeger A, Fröhlich M, Klum S, Lantow M, Viergutz T, Weiss DG, Kriehuber R (2015). Characterization of apoptosis signaling cascades during the differentiation process of human neural rencell VM progenitor cells in vitro. Cellular and Molecular Neurobiology.

[bib26] Kim M, Kim T, Johnson RL, Lim DS (2015). Transcriptional co-repressor function of the hippo pathway transducers YAP and TAZ. Cell Reports.

[bib27] Leverson JD, Zhang H, Chen J, Tahir SK, Phillips DC, Xue J, Nimmer P, Jin S, Smith M, Xiao Y, Kovar P, Tanaka A, Bruncko M, Sheppard GS, Wang L, Gierke S, Kategaya L, Anderson DJ, Wong C, Eastham-Anderson J, Ludlam MJ, Sampath D, Fairbrother WJ, Wertz I, Rosenberg SH, Tse C, Elmore SW, Souers AJ (2015). Potent and selective small-molecule MCL-1 inhibitors demonstrate on-target cancer cell killing activity as single agents and in combination with ABT-263 (navitoclax). Cell Death & Disease.

[bib28] Lin L, Sabnis AJ, Chan E, Olivas V, Cade L, Pazarentzos E, Asthana S, Neel D, Yan JJ, Lu X, Pham L, Wang MM, Karachaliou N, Cao MG, Manzano JL, Ramirez JL, Torres JM, Buttitta F, Rudin CM, Collisson EA, Algazi A, Robinson E, Osman I, Muñoz-Couselo E, Cortes J, Frederick DT, Cooper ZA, McMahon M, Marchetti A, Rosell R, Flaherty KT, Wargo JA, Bivona TG (2015). The Hippo effector YAP promotes resistance to RAF- and MEK-targeted cancer therapies. Nature Genetics.

[bib29] Meier P, Finch A, Evan G (2000). Apoptosis in development. Nature.

[bib30] Morin-Kensicki EM, Boone BN, Howell M, Stonebraker JR, Teed J, Alb JG, Magnuson TR, O'Neal W, Milgram SL (2006). Defects in yolk sac vasculogenesis, chorioallantoic fusion, and embryonic axis elongation in mice with targeted disruption of Yap65. Molecular and Cellular Biology.

[bib31] Nemazee D (2017). Mechanisms of central tolerance for B cells. Nature Reviews Immunology.

[bib32] Oltersdorf T, Elmore SW, Shoemaker AR, Armstrong RC, Augeri DJ, Belli BA, Bruncko M, Deckwerth TL, Dinges J, Hajduk PJ, Joseph MK, Kitada S, Korsmeyer SJ, Kunzer AR, Letai A, Li C, Mitten MJ, Nettesheim DG, Ng S, Nimmer PM, O'Connor JM, Oleksijew A, Petros AM, Reed JC, Shen W, Tahir SK, Thompson CB, Tomaselli KJ, Wang B, Wendt MD, Zhang H, Fesik SW, Rosenberg SH (2005). An inhibitor of Bcl-2 family proteins induces regression of solid tumours. Nature.

[bib33] Opferman JT (2008). Apoptosis in the development of the immune system. Cell Death & Differentiation.

[bib34] Preta G, Fadeel B (2012). Scythe cleavage during Fas (APO-1)-and staurosporine-mediated apoptosis. FEBS Letters.

[bib35] Rosenbluh J, Nijhawan D, Cox AG, Li X, Neal JT, Schafer EJ, Zack TI, Wang X, Tsherniak A, Schinzel AC, Shao DD, Schumacher SE, Weir BA, Vazquez F, Cowley GS, Root DE, Mesirov JP, Beroukhim R, Kuo CJ, Goessling W, Hahn WC (2012). β-Catenin-driven cancers require a YAP1 transcriptional complex for survival and tumorigenesis. Cell.

[bib36] Sarosiek KA, Ni Chonghaile T, Letai A (2013). Mitochondria: gatekeepers of response to chemotherapy. Trends in Cell Biology.

[bib37] Schlegelmilch K, Mohseni M, Kirak O, Pruszak J, Rodriguez JR, Zhou D, Kreger BT, Vasioukhin V, Avruch J, Brummelkamp TR, Camargo FD (2011). Yap1 acts downstream of α-catenin to control epidermal proliferation. Cell.

[bib38] Song S, Honjo S, Jin J, Chang SS, Scott AW, Chen Q, Kalhor N, Correa AM, Hofstetter WL, Albarracin CT, Wu TT, Johnson RL, Hung MC, Ajani JA (2015). The hippo coactivator YAP1 Mediates EGFR overexpression and confers chemoresistance in esophageal cancer. Clinical Cancer Research.

[bib39] Souers AJ, Leverson JD, Boghaert ER, Ackler SL, Catron ND, Chen J, Dayton BD, Ding H, Enschede SH, Fairbrother WJ, Huang DC, Hymowitz SG, Jin S, Khaw SL, Kovar PJ, Lam LT, Lee J, Maecker HL, Marsh KC, Mason KD, Mitten MJ, Nimmer PM, Oleksijew A, Park CH, Park CM, Phillips DC, Roberts AW, Sampath D, Seymour JF, Smith ML, Sullivan GM, Tahir SK, Tse C, Wendt MD, Xiao Y, Xue JC, Zhang H, Humerickhouse RA, Rosenberg SH, Elmore SW (2013). ABT-199, a potent and selective BCL-2 inhibitor, achieves antitumor activity while sparing platelets. Nature Medicine.

[bib40] Stein C, Bardet AF, Roma G, Bergling S, Clay I, Ruchti A, Agarinis C, Schmelzle T, Bouwmeester T, Schübeler D, Bauer A (2015). YAP1 exerts its transcriptional control via TEAD-mediated activation of enhancers. PLOS Genetics.

[bib41] Tao ZF, Hasvold L, Wang L, Wang X, Petros AM, Park CH, Boghaert ER, Catron ND, Chen J, Colman PM, Czabotar PE, Deshayes K, Fairbrother WJ, Flygare JA, Hymowitz SG, Jin S, Judge RA, Koehler MF, Kovar PJ, Lessene G, Mitten MJ, Ndubaku CO, Nimmer P, Purkey HE, Oleksijew A, Phillips DC, Sleebs BE, Smith BJ, Smith ML, Tahir SK, Watson KG, Xiao Y, Xue J, Zhang H, Zobel K, Rosenberg SH, Tse C, Leverson JD, Elmore SW, Souers AJ (2014). discovery of a potent and selective BCL-XL inhibitor with in vivo activity. ACS Medicinal Chemistry Letters.

[bib42] Wang ES, Reyes NA, Melton C, Huskey NE, Momcilovic O, Goga A, Blelloch R, Oakes SA (2015). Fas-activated mitochondrial apoptosis culls stalled embryonic stem cells to promote differentiation. Current Biology.

[bib43] Xu W, Jing L, Wang Q, Lin CC, Chen X, Diao J, Liu Y, Sun X (2015). Bax-PGAM5L-Drp1 complex is required for intrinsic apoptosis execution. Oncotarget.

[bib44] Yamane T, Dylla SJ, Muijtjens M, Weissman IL (2005). Enforced Bcl-2 expression overrides serum and feeder cell requirements for mouse embryonic stem cell self-renewal. PNAS.

[bib45] Zhao J, Li X, Yang Y, Zhu D, Zhang C, Liu D, Wu K, Zhao S (2016). Effect of YAP1 silencing on esophageal cancer. OncoTargets and Therapy.

