# Supplementary material for: Yap1 safeguards mouse embryonic stem cells from excessive apoptosis during differentiation
Source: eLife. 2018 Dec 18;7:e40167. doi: 10.7554/eLife.40167 (PMC6307859; doi:10.7554/eLife.40167)
Supplement: Supplementary file 1. — Table of RT-qPCR primers used for qPCR gene expression assays in this study. Primers were designed using Primer3 and verified by melt curve analysis. Supplementary Table S2. Table of cloning primers used for dual luciferase assay including chromosome coordinates (using mm9) and regulatory element length. Supplementary Table S3. Table of shRNA and siRNA used in KD experiments including target, ID, and sequence or target position. [file elife-40167-supp1.docx]

**Supplemental Tables (Tables S1-S3)**

**Table S1. RT-qPCR Primers Used in this Study**

| **Primer Name** | **Sequence** |
| --- | --- |
| Yap1_RT_F | GAGCAAGCCATGACTCAGGA |
| Yap1_RT_R | TCTGGAGACCATGTTTCTGAAGT |
| Bcl2_RT_F | TGTGGATGACTGAGTACCTGAAC |
| Bcl2_RT_R | GAGAAATCAAACAGAGGTCGCATG |
| BclxL_RT_F | AAGGAGATGCAGGTATTGGTGAG |
| BclxL_RT_R | CATTGTTCCCGTAGAGATCCACA |
| Mcl1_RT_F | ATCGAACCATTAGCAGAAACTATCA |
| Mcl1_RT_R | TCTAGGTCCTGTACGTGGAAGAA |
| PumaRD_RT_F | AGTGCGTGTGGAGGAGGAGGAGT |
| PumaRD_RT_R | GTCGGTGTCGATGCTGCTCTTCT |
| Noxa_RT_F | GAAGTCGCAAAAGAGCAGGATGAG |
| Noxa_RT_R | AATTCACTTTGTCTCCAATCCTCCG |
| Bmf_RT_F | GTTCTTCACCCTGGACCCTG |
| Bmf_RT_R | CTAGCTCCTCCACACACTGAG |
| Fas_RT_F | AACCATTATGCTGATAAATGCAGAAGA |
| Fas_RT_R | CAGGAGAATCGCAGTAGAAGTCT |
| FasL_RT_F | TTAGGAATGTATCAGCTCTTCCACC |
| FasL_RT_R | TACTTTAAGGCTTTGGTTGGTGAAC |
| Nanog_RT_F | AGGGTCTGCTACTGAGATGCTCTG |
| Nanog_RT_R | CAACCACTGGTTTTTCTGCCACCG |
| Nes_RT_F | AGGACCAGGTGCTTGAGAGA |
| Nes_RT_R | TTCGAGAGATTCGAGGGAGA |
| Otx2_RT_F | AAGTGAGTTCAGAGAGTGGAACAAG |
| Otx2_RT_R | CTCCAGATAGACACTGGAGCACT |
| Gbx2_RT_F | AAGACGAGTCAAAGGTGGAAGAT |
| Gbx2_RT_R | CAGTCTGACCAGGCAAATTGT |
| Gata4_RT_F | TTCTCAGAAGGCAGAGAGTGTGT |
| Gata4_RT_R | ATGCCGTTCATCTTGTGATAGAG |
| Gata6_RT_1_F | GACGGCACCGGTCATTACC |
| Gata6_RT_1_R | ACAGTTGGCACAGGACAGTCC |
| Sox17_RT_F | CTAAGCAAGATGCTAGGCAAGTCT |
| Sox17_RT_R | GTACTTGTAGTTGGGGTGGTCCT |
| Casp2_RT_F | TTATCACTTTGGAAATGAGGGAGCT |
| Casp2_RT_R | TTCACAGAAGGCATCAAAAGCC |
| Casp3_RT_F | GATCTTACTCGTGAAGACATTTTGGAA |
| Casp3_RT_R | CTTCATCACCATGGCTTAGAATCAC |
| Casp6_RT_F | TCATCTTCAATCACGAGAGGTTCTT |
| Casp6_RT_R | CAAATCCTAGATCTGAAAACCTGCG |
| Casp7_RT_F | CGCTCCTCTATCATCTCCTCTATTC |
| Casp7_RT_R | GAAATCCATGCGGTACAGATAAGTG |
| Casp8_RT_F | CTGGTCAACTTCCTAGACTGCAA |
| Casp8_RT_R | ATCTCAATTCCAACTCGCTCACT |
| Casp9_RT_F | CTCAGACCAGAAACACCCAGG |
| Casp9_RT_R | GAATCCAGGGTGTATGCCATATCT |
| Cdx2_RT_F | GCGAAACCTGTGCGAGTGGATG |
| Cdx2_RT_R | CGGTATTTGTCTTTTGTCCTGGTTTTCA |
| Gata3_RT_F | GTAGTGCCCGGTACCATCTC |
| Gata3_RT_R | CTACCGGGTTCGGATGTAAGT |
| Gsc_RT_F | AGAAGGTGGAGGTCTGGTTTAAG |
| Gsc_RT_R | GAGGACGTCTTGTTCCACTTCT |
| T_RT_F | CTTCAAGGAGCTAACTAACGAGATG |
| T_RT_R | GTCCAGCAAGAAAGAGTACATGG |
| Wnt3_RT_F | CTATGAACAAGCACAACAATGAAGC |
| Wnt3_RT_R | TTTAGGTGCATGTGGTCCAGG |
| Dnmt3b_RT_F | ACTTCAGTGACCAGTCCTCAG |
| Dnmt3b_RT_R | CTGCGTGTAATTCAGAAGGCT |
| Fgf5_RT_F | CAAAGTCAATGGCTCCCACGAA |
| Fgf5_RT_R | CTACAATCCCCTGAGACACACAGC |

**Table S2. Primers Used for the Luciferase Assay**

| **Region Name** | **Start** | **Forward Oligo** | **Reverse Oligo** | **Length** |
| --- | --- | --- | --- | --- |
| Mcl1 Distal | chr3:95456899 | TACGCGTGCTAGCCCGGGgcattgaggacgctgatgaag | GCAGATCTCGAGCCCGGGgtttaacacaaccctggcagtc | 279 |
| Mcl1 Distal ΔTBS frg 1 | chr3:95456899 | CCGAGCTCTTACGCGTGCTAGCgcattgaggacgctgatgaagg | gcctctcctcctgctgtgctttaaggcatgtgccaccgct | 181 |
| Mcl1 Distal ΔTBS frg 2 | chr3:95457061 | agcggtggcacatgccttaaagcacagcaggaggagaggc | GATGCAGATCGCAGATCTCGAGgtttaacacaaccctggcagtc | 93 |
| Bcl2 Intronic | chr1:108460969 | TACGCGTGCTAGCCCGGGgaaggactcagttaatacccacgag | GCAGATCTCGAGCCCGGGgcaaactcagatcatcatgctaaaatg | 419 |
| Bcl2 Peak 4 (for Tandem) | chr1:108526701 | cattttagcatgatgatctgagtttgcgaaccaagggggcagcttaacg | GATGCAGATCGCAGATCTCGAGattactgattatcctctggttc | 277 |
| BclxL Intronic | chr2:152618090 | TACGCGTGCTAGCCCGGGctactgggaagaggaagaaaggaac | GCAGATCTCGAGCCCGGGgttagctgttctagacttcgtggta | 628 |
| Bmf Intronic | chr2:118369993 | TACGCGTGCTAGCCCGGGtgtcttgtggctttgctaggtag | GCAGATCTCGAGCCCGGGctgggtgtgtttaagagttttggtc | 748 |
| Noxa Downstream | chr18:66634965 | TACGCGTGCTAGCCCGGGcaaagtcaaaatgcaggccca | GCAGATCTCGAGCCCGGGgcttatatgtgctctgacgaca | 660 |
| Puma Intronic | chr7:16898735 | TACGCGTGCTAGCCCGGGaaataggggtacttaggtcggataa | GCAGATCTCGAGCCCGGGaatgaaggagatggaggactttg | 1169 |

*Capital letters denote sequence homology to pGL3-promoter whereas lower case letters denote regulatory element sequence.

**Table S3. Table S3. shRNA Constructs and siRNA Duplexes Used in This Study**

| **Gene** | **TRC # (shRNA) or ID (siRNA)** | **Sequence (shRNA) or mRNA Target Position (siRNA)** | **KD** |
| --- | --- | --- | --- |
| Yap1 | TRCN0000238433 | CCGGTCCAACCAGCAGCAGCAAATACTCGAGTATTTGCTGCTGCTGGTTGGATTTTTG | 1 |
| Yap1 | TRCN0000238434 | CCGGACTTGGAGGCGCTCTTCAATGCTCGAGCATTGAAGAGCGCCTCCAAGTTTTTTG | 2 |
| Casp9 | TRCN0000320959 | CCGGCCACTGCCTCATCATCAACAACTCGAGTTGTTGATGATGAGGCAGTGGTTTTTG | 1 |
| Casp9 | TRCN0000321028 | CCGGAGAGGTTCTCAGACCAGAAACCTCGAGGTTTCTGGTCTGAGAACCTCTTTTTTG | 2 |
| Bcl2 | TRCN0000226262 | CCGGCCACAAGTGAGGTCGACAAACCTCGAGGTTTGTCGACCTCACTTGTGGTTTTTG | 1 |
| Bcl2 | TRCN0000226261 | CCGGCATGCGACCTCTGTTTGATTTCTCGAGAAATCAAACAGAGGTCGCATGTTTTTG | 2 |
| Bmf | TRCN0000009715 | CCGGCATCGGCTTCATACGCAACAACTCGAGTTGTTGCGTATGAAGCCGATGTTTTT | 1 |
| Bmf | TRCN0000009717 | CCGGGCTAGAAGATGATGTGTTCCACTCGAGTGGAACACATCATCTTCTAGCTTTTT | 2 |
| Bbc3  (Puma) | TRCN0000271164 | CCGGAGCCCAACTAGGTGCCTACACCTCGAGGTGTAGGCACCTAGTTGGGCTTTTTTG | 1 |
| Bbc3  (Puma) | TRCN0000271167 | CCGGCAAGAAGAGCAGCATCGACACCTCGAGGTGTCGATGCTGCTCTTCTTGTTTTTG | 2 |
| Mcl1 | SASI_Mm02_00314161 | 1424 | 1 |
| Mcl1 | SASI_Mm01_00048594 | 924 | 2 |
| Bcl2l1  (BclxL) | SASI_Mm02_00316924 | 707 | 1 |
| Bcl2l1  (BclxL) | SASI_Mm02_00316925 | 658 | 2 |
